# Climate change-induced stress in the honey bee *Apis mellifera* L.- a genetic review

**DOI:** 10.3389/fphys.2025.1623705

**Published:** 2025-09-17

**Authors:** Soledad Sagastume, Giovanni Cilia, Dora Henriques, Carlos Yadró, Miguel Corona, Mariano Higes, M. Alice Pinto, Antonio Nanetti, Raquel Martín-Hernández

**Affiliations:** ^1^ Laboratorio de Patología Apícola, Centro de Investigación Apícola y Agroambiental de Marchamalo, (CIAPA), Instituto Regional de Investigación y Desarrollo Agroalimentario y Forestal de Castilla-La Mancha (IRIAF), Marchamalo, Spain; ^2^ CREA Centro di Ricerca Agricoltura e Ambiente (CREA-AA), Bologna, Italy; ^3^ Centro de Investigação de Montanha (CIMO), LA SusTEC, Instituto Politécnico de Bragança, Bragança, Portugal; ^4^ Honey Bee Research Laboratory, Agricultural Research Service, United States Department of Agriculture, Beltsville, MD, United States

**Keywords:** climate change, honey bee, *Apis mellifera* subspecies, stress, stress-related genes, HSPs, epigenetics, stress response

## Abstract

Climate change is a powerful driver of stress, as it reinforces hotter and drier environments. For bees, the most concerning aspects of these new environmental conditions are the resistance and resilience of bees to changes in temperature, humidity and ultraviolet radiation, as well as the negative effect on diversity of food resources which can lead in nutritional stress. The climatic vulnerability of various bee species and subspecies varies worldwide, as they experience varying levels of stress and display distinct behaviors, weaknesses, and lifespans. To understand these differences, it is crucial to consider both the genetics and epigenetics of bees, as these factors play a key role in their response, resistance, and adaptation to new stressors. This review provides a guide of genetic and epigenetic markers involved in the cellular response of *Apis mellifera* to most common stressors derived from climate change. Understanding how the various molecular mechanisms interact to restore homeostasis during the stress response is essential for designing future studies based on molecular markers.

## 1 Introduction

Honey bees are social insects of great ecological and economic importance, but they have experienced substantial losses over the last years due to the action of several interacting biotic and abiotic stressors. These include invasive alien predators, parasites and pathogens, pesticides, and climate change [reviewed by Zhao et al. (2021), Even et al. (2012)]. One of the main impacts of climate change is the intensification of extreme weather events, such as heavy rainfall, prolonged droughts, and heat waves (IPCC-Intergovernmental Panel on Climate Change, 2007), which may severely impact honey bees’ wellbeing. On one hand, these events can indirectly affect honey bee nutrition by disrupting the availability of floral resources and reducing the quantity and quality of nectar and pollen [reviewed by Obeso and Herrera (2018)]. On the other hand, they can directly cause heat stress on honey bees, impairing their foraging efficiency and metabolic functions [reviewed by Zhao et al. (2021)]. Whether honey bees are able to adapt and survive changes in temperature, humidity, and ultraviolet (UV) radiation, and to a reduction in the quantity and diversity of food resources, is a question of utmost importance. However, the vulnerability of bees to climatic vagancies is not necessarily the same worldwide. Even in the same environment, different bee species and subspecies may suffer different stress levels and exhibit different behaviours, weaknesses and lifespans [reviewed by Zhao et al. (2021)]. This would mean that bee species and subspecies may differ in their ability to adapt, resist and be resilient to environmental changes.

Genetic variability is key to resistance and adaptation in stressful environments. Several candidate Single Nucleotide Polymorphisms (SNPs) mapped to genes involved in reproduction, immunity, olfaction, circadian rhythm, lipids biosynthesis and storage were linked to local adaptation in the western honey bee *Apis mellifera* (Chávez-Galarza et al., 2013; Wallberg et al., 2014; Chen et al., 2016; Henriques et al., 2018). However, beyond genetic determination, complex interactions between genetic and epigenetic factors are known to shape the diversity of organismal phenotypes (West-Eberhard, 2003). A phenotype can change in response to environmental signals without altering its genotype, resulting in modifications to an organism’s physiology and behaviour. This ability is called “phenotypic plasticity” and is remarkably developed in *A. mellifera*, which can respond to environmental cues to generate dramatically distinct phenotypes, such as queens or workers, through nutritional stimuli such as royal jelly (Winston, 1991; Corona et al., 2016). Interestingly, the *A. mellifera* genome has been described as structured with respect to plasticity, where stress-related genes are organised into clusters that show coordinated gene expression in response to environmental changes (Duncan et al., 2020). In addition to epigenetics, RNA processing mechanisms are also involved in phenotypic plasticity; splicing is the process by which introns are removed from a gene’s primary transcript, resulting either in a single functional protein (constitutive splicing) or in various structurally and functionally isoforms (alternative splicing). Alternative splicing (AS) can be triggered by physiological needs and environmental stimuli, often representing a primary source of phenotypic diversity within the proteome of eukaryotic cells [reviewed by Ast (2004), Blencowe (2006), Maniatis and Tasic (2002)]. It also plays a key role in cellular stress tolerance [reviewed by Biamonti and Caceres (2009)].

**FIGURE 1:**
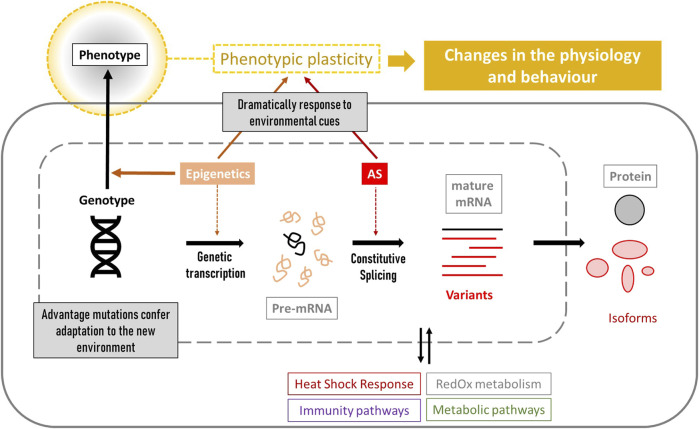
Overview of the genetic and cellular processes that lead to phenotypic changes. Cellular biomarkers include genes related to stress resistance (genotypes and gene-expression levels), epigenetic factors influencing gene expression, detection of mRNA variants produced by alternative splicing (AS), and the study of the corresponding synthetized proteins and their role in the different stress-related pathways such as heat shock response, immunity, metabolism and RedOx homeostasis. All of these characteristics confer different phenotypic plasticity to each individual.

Genetic studies are a valuable tool to understand the effects of stressors on different molecular mechanisms. This review proposes genetic markers for *Apis mellifera* focusing mainly on climate change stressors such as temperature, humidity, UV exposure and food scarcity. These markers belong to different molecular mechanisms summarized in Figure 1, ranging from genetics to epigenetics, and affecting an organism´s phenotype. Thus, it can serve as a basis for designing new studies that consider the relationship with the stressor(s), as well as the type of gene expression (constitutive or inducible), splicing-related processes (key in PCR designs based on RNA) and epigenetic factors that might affect gene expression. In addition, the knowledge of the role of each marker in the corresponding cellular pathways will help future studies to select accurate molecular markers and correctly interpret the results.

## 2 Methods

This review was based on a published bibliography of original articles, reviews, book chapters and web pages obtained from scientific sources such as PubMed (PubMed, 2025), Scopus (Scopus, 2025), Google Scholar (Google Scholar, 2025), and Web of Science (Web Of Science, 2025). Candidate search terms are shown in Supplementary Material (Supplementary Table S1).

**FIGURE 2:**
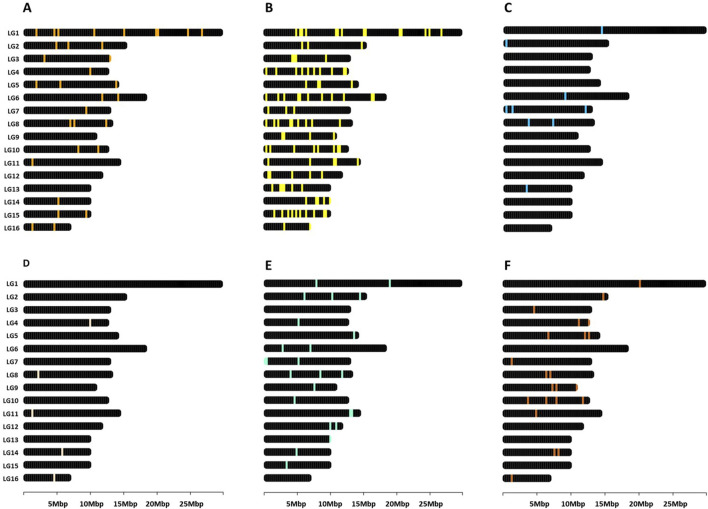
Genomic map in *Apis mellifera*. Distribution of genes related to **(A)** heat shock response (Supplementary Table S2), **(B)** other heat stress-related pathways (Supplementary Table S3), **(C)** humidity (Supplementary Table S4), **(D)** Ultraviolet exposure (Supplementary Table S5), **(E)** nutrition (Supplementary Table S6), and **(F)** epigenetics (Supplementary Table S7). The figures were created using the R package chromoMap v0.4.1 (Anand and Rodriguez Lopez, 2022). The chromosomes were annotated using the honey bee reference genome Amel_HAv3.1.

Gene descriptions and nomenclature follow the conventions established by NCBI. Given that the *Apis mellifera* genome has been characterized as organized in clusters associated with phenotypic plasticity (Duncan et al., 2020), the genetic markers referenced throughout this study were visualized as loci mapped onto their respective chromosomes (Figure 2). This was accomplished using the R package chromoMap v0.4.1 (Anand and Rodriguez Lopez, 2022). Such spatial representation of genomic features may prove instrumental for future research, particularly when selecting genetic markers based on their chromosomal location.

## 3 What is stress for honey bees?

Although the term “stress” is commonly understood to have a negative connotation, stress is a natural state pervasive in all known biological systems. These systems maintain a complex dynamic equilibrium called homeostasis that is vulnerable to destabilization in the presence of stressors. After many years of controversy, stress is defined as a “state of homeostasis being challenged,” and stressors as “the factors with the potential to directly challenge homeostasis” [reviewed by Lu et al. (2021)]. An optimal stress level plays a key role in the health and adaptability of organisms. Depending on the level of exposure to the stressor(s), stress can be classified as: (i) distress (or bad stress), which occurs when the state of homeostasis is severely challenged by high levels of stress, inducing a severe response that impairs homeostasis and threatens health; (ii) sustress (or inadequate stress), which is the consequence of inappropriate stressor effects that do not challenge homeostasis but undermine its capacity, and threaten health, and (iii) eustress (or good stress), in which the system is mildly challenged by moderate levels of stressors, inducing a mild response, enhancing the buffering capacity of homeostasis and benefiting health [reviewed by Lu et al. (2021)]. Previous terminology defined “physiological” stress as encompassing environmental stress, intrinsic developmental stress and ageing [reviewed by Kagias et al. (2012)], initially understood as eustress. However, if an organism is unable to cope with one or more of these stressors, the level of stress increases (distress) and becomes pathological.

Regardless of its type or intensity, stress has various consequences at different levels. In honey bees, these consequences can be observed in their behavioral, physiological, and cellular responses. The first consists of the term “fight-or-flight.” For example, sting extension has been used to evaluate sensitivity to stressors in honey bees. This behaviour is widely considered to be indicative of stress as well as an aggressive response. Physiological responses include hormone and neurotransmitter levels. Finally, cellular responses consist of the activation of several molecular mechanisms to control the stressful state and restore the damage. Perhaps the best known and most studied cellular response is the production of stress-related proteins, such as Heat Shock Proteins (HSPs) and antioxidant enzymes, known as stress biomarkers (review by Even et al. (2012)]. At all these levels, cells and systems use different strategies to respond and adapt to environmental changes. Suppose these strategies are effective in coping with the stressful situation. In this case, the organism would undergo a process of adaptation, enabling it to survive and reproduce under new conditions.

## 4 Temperature

Rising temperature is one of the main effects of climate change (IPCC-Intergovernmental Panel on Climate Change, 2007) and is of concern because temperature is one of the most stressful abiotic stresses on living organisms. But in addition to the effects of temperature on individual organisms, the consequences for an entire biocenosis depend on how each organism changes to adapt. For example, changes in temperature are affecting the hibernation cycle of honey bees (which would affect pollination, and hence plant reproduction), but also the timing and amount of flowering (which changes the availability of food resources for bees) (Obeso and Herrera, 2018). This example is very important because nutritional stress is an important indirect stressor to honey bees from rising temperatures [reviewed by Quinlan and Grozinger (2023)] and is discussed in section 7 of this review.

Focusing only on honey bees, the effect of temperature must be analysed from two perspectives: as a super-organism (colony) and at the individual level (single bee). In both cases, honey bees have developed mechanisms to resist stress and restore homeostasis. As a super-organism, they can regulate the temperature inside the colony, maintaining it constant at approximately 35 °C. In a cold environment, all the bees cluster around the queen and brood to generate and maintain heat (Southwick, 1985; Southwick and Heldmaier, 1987). In a warm environment, the colony employs a combination of ventilation, achieved through coordinated wing fanning by honey bees at hive entrance (at a rate of up to 60 L/min), and evaporative cooling, achieved through the use of water from the bee´s bodies to create droplets that cool down the nest (Southwick and Heldmaier, 1987; Jones and Oldroyd, 2006). All these actions at the community level require also actions at the individual level. Here, cellular mechanisms are key to coping with temperature changes.

Heat stress negatively impacts key biological processes in honey bees, including physiological and behavioral development and immunocompetence (Bordier et al., 2017; Medina et al., 2018; Alqarni, 2020; Greenop et al., 2020). It is worth mentioning that the impact of heat stress on honey bees and their defence mechanisms vary across species and even subspecies [reviewed by Abou-Shaara et al. (2017), Zhao et al. (2021)]. In *Apis mellifera*, two of the 31 currently recognized subspecies (Ruttner, 1988; De la Rúa et al., 2009), *A. mellifera ligustica* (native to Italy) and *A.m*. *carnica* (native to the Balkan region), have been the focus of several comparative studies due to their widespread commercial use beyond their native ranges. A study on thermal limits and metabolic rates showed that *A .m. ligustica* foragers are more tolerant to high temperatures than *A. m. carnica* (Kovac et al., 2007), suggesting that the Italian honey bee is better fit for warmer climates. In Saudi Arabia, several studies have compared the two commercial subspecies with the native *A. m*. *jemenitica*, which evolved in semi-arid and desert environments. Interestingly, all the studies showed that *A. m. ligustica* and *A. m. carnica* had lower heat tolerance and survival rates than *A. m. jemenitica* (Abou-Shaara et al., 2012; Alattal et al., 2015; Alqarni et al., 2019). In another study comparing the native subspecies of Algeria, *A. m. sahariensis* and *A. m. intermissa*, a differential response to heat stress was also found. Interestingly, *A.m. sahariensis,* the subspecies adapted to the desert environment with extreme temperatures, reacted better to heat than *A.m. intermissa*, the subspecies native to the southern shore of the Mediterranean, where the average temperatures are comparatively milder (Khedidji et al., 2024). As for interspecific variability, a study in China showed a higher temperature adaptability in *Apis cerana* than in *A. mellifera*. However, the contrary was observed on survival assays; under constant heat and humidity, *A. mellifera* showed higher survival rates than *A. cerana* (Li et al., 2019). These findings suggest that variations within the genus *Apis* must be studied to understand the different thermotolerances and metabolic responses to heat stress, as well as the expected differential transcriptional regulation. However, there are still many honey bee species and subspecies for which no information is available.

### 4.1 Heat shock response

**FIGURE 3:**
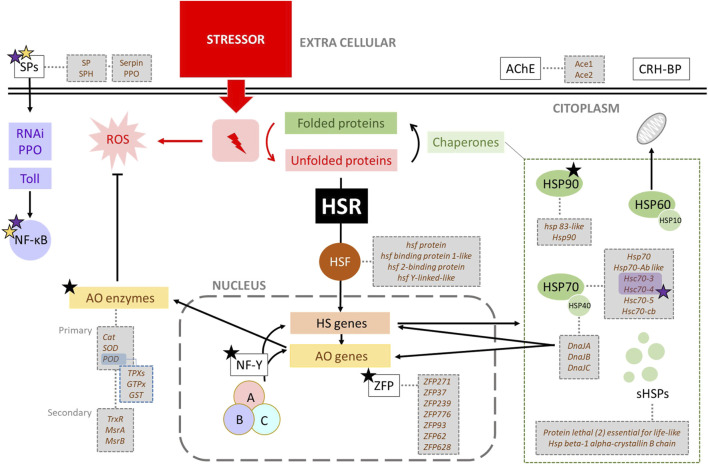
General outline of heat stress in *Apis mellifera* showing related genes. One stressor, like heat, triggers the heat shock response (HSR) when proteins denaturation occurs. The HSR starts with the activation of Heat shock Factor (HSF), a transcription factor that induces the expression of heat shock (HS) genes. These genes encode various chaperones (HSP90, HSP70, HSP60 or chaperonines, HSP40 and small HSPs), which assist in refolding denatured proteins. Specially HSP40 (DnaJ) also regulates the expression of many antioxidant and HS genes. Stressors typically generate ROS, whose levels are controlled by antioxidant enzymes (AO). The genes encoding these enzymes can be activated by the presence of ROS, the action of DnaJ proteins (HSP40), the Nuclear Factor-Y (NF-Y), and Serine Proteases (SPs). Black, purple and yellow stars indicate alternative splicing (AS), involvement in immune pathways, and relationship with antioxidant metabolism, respectively.

Heat is a highly negative stressor that alters important cellular structures and mechanisms, including actin filament organisation, protein aggregation, disruption of intracellular transport, fragmentation of the Golgi and endoplasmic reticulum, altering membrane-bound organelles, RNA splicing, ribosomal activity and translation, and ultimately leading to cell cycle arrest and stagnation of growth and proliferation [reviewed by Richter et al. (2010), Morimoto (2011)]. Due to its highly detrimental impact on cell integrity, heat triggers a quick and robust heat shock response (HSR) at the molecular level. This response involves the activation of heat shock genes encoding heat shock proteins (HSPs), antioxidant metabolism genes, and genes associated with alternative pathways related to heat stress (Figure 3). Activation of the HSR enhances cellular resistance to stress, a phenomenon known as thermotolerance [reviewed by Calderwood and Ciocca (2008)]. Thermotolerance is defined as the temperature range between the lowest temperature (critical thermal minimum, CTmin) and the highest temperature (critical thermal maximum, CTmax) at which an organism can maintain muscle control [reviewed by Perez and Aron (2020)]. Consequently, thermal tolerance and its degree of plasticity play key roles in determining the geographical distribution of species.

However, the HSR is not triggered by heat *per se* but by the presence of unfolded proteins that result from a variety of stresses (Figure 3), including oxidative stress, heavy metals, ethanol or other toxic substances [reviewed by Richter et al. (2010)]. Therefore, some of the genes related to thermal stress will also be involved in another kind of resistance or adaptation associated with different stressors. On the other hand, the AS mechanism is also important in the HSR (Fujikake et al., 2005; Ruan et al., 2015) due to the generation of new and different isoforms relevant to function under new stressful conditions.

The temperatures experienced by honey bees during their normal activity result in a strong induction of HSR (McKinstry et al., 2017). The HSR usually starts with the activation of *Hsp* genes through transcriptional factors, the heat shock factor (HSF) family, which specifically bind to the heat shock element (HSE) in the promoter region of the *Hsp* genes, and regulate their activity in normal conditions [reviewed by Morimoto (2011), Garbuz (2017). Therefore, the HSF family is considered a transcriptional activator of all significant *Hsp* genes, and it is required in the regulation of various environmental stresses [reviewed by Zhang et al. (2011), Wu (1995), Zou et al. (1998)]. Four *Hsf-*related genes have been described in *A. mellifera* (Supplementary Table S2), and changes in their expression have been observed in honey bees suffering from heat stress (McKinstry et al., 2017; Al-Ghzawi et al., 2022).

#### 4.1.1 Heat shock proteins

The key to HSR lies in the heat shock proteins (HSPs), which are proteins that protect or restore cellular structures or components that are prone to damage by heat shock. Not surprisingly, there is a broad functional classification of these stress-inducible proteins, such as molecular chaperones, components of the proteolytic system, RNA- and DNA-modifying enzymes, metabolic enzymes, regulatory proteins (like transcription factor or kinases), proteins involved in sustaining cellular structures such as cytoskeleton, and finally, transport, detoxifying, and membrane-modulation proteins (Richter et al., 2010). Under moderate stress, the synthesis of HSPs is the basis of resistance to such stress and may even provide “cross-protection” against other types of stress (Lindquist, 1986).

The most well-known HSPs are the molecular chaperones, both because they were the first to be discovered and because they are the most studied in relation to thermal stress. Their primary function is to assist in the folding and unfolding of proteins, and they are usually classified according to their molecular weight: HSP100 (78–104 kDa), HSP90 (82–96 kDa), HSP70 (68–78 kDa), HSP60 or chaperonins (60 kDa), HSP40 or DNAj domain proteins (40 kDa) and small HSPs (sHSPs) or α-crystallin proteins (10–30 kDa) [reviewed by Garbuz (2017)]. Another classification of HSPs is based on the pattern of gene expression of the corresponding genes, which may be inducible or constitutive. Inducible *Hsp* genes are typically expressed at extremely low levels, but under stress, their transcription intensity increases by a factor of several hundred or several thousand relative to baseline. Contrarily, constitutive *Hsp* genes are expressed at relatively high levels even under normal temperatures (they are supposed to possess a physiological function), and their transcription increases only several-fold under stress (Pardue et al., 1980). In honey bees, HSPs seem to serve a significant role in their responses to abiotic stressors (Table 1), like high and low temperature, dehydration, UV radiation, and starvation (Kim et al., 2019). The most important HSPs involved in HSR in *A. mellifera* are HSP90, HSP70, HSP60, HSP40, and small HSPs (sHSPs) [reviewed by Abou-Shaara (2024)].

**TABLE 1:** Genetic marker groups, their relationship to stress factors and immunity, and the characteristics of their gene expression. Grey letters indicate an indirect relationship through another stressor (humidity influences directly the heat stress degree, and hence, the corresponding molecular response). In the gene expression pattern, “C” and “I” stand for constitutive and inducible, respectively. “Id” indicates ‘indeterminate’ for those markers that are considered inducible or constitutive depending on the context.

Abbreviation	Description	Stressor					Gene expression	
		Temperature	Humidity	UV radiation	Nutrition	Immunity	Pattern	mRNA variants
HSF	Heat shock factor proteins	**x**	**x**				Id	
HSP90	Heat shock proteins 90 kDa	**x**	**x**				C	**x**
HSP70	Heat shock proteins 70 kDa	**x**	**x**				I	
HSC70	Heat shock proteins 70 kDa- cognate forms	**x**	**x**			**x**	C	**x**
HSP60	Heat shock protein 60 kDa	**x**	**x**				Id	
HSP40	Heat shock proteins 40 kDa	**x**	**x**	**x**			Id	
HSP10	Heat shock protein 10 kDa	**x**	**x**				Id	
sHSP	Small Heat shock proteins	**x**	**x**	**x**			Id	
NF-Y	Nuclear factor Y subunits	**x**	**x**	**x**			Id	**x**
ZFP	Zinc finger proteins	**x**	**x**	**x**			Id	**x**
SP	Serine proteases	**x**	**x**	**x**		**x**	Id	
Ache	Acetylcholinesterase	**x**	**x**	**x**			Id	
Crh-BP	Corticotropin-releasing hormone-binding protein	**x**	**x**				Id	
AO	Antioxidant enzymes	**x**	**x**	**x**	**x**	**x**	Id	**x**
Pla2	Phospholipase A2		**x**			**x**	Id	
Afp	Antifreeze protein	**x**	**x**				Id	
PPO	Prophenoloxidase	**x**	**x**	**x**		**x**	Id	
FOXO	Forkhead box protein sub-group O				**x**		Id	
ILP	Insulin-like peptides				**x**		Id	
IGF	Insulin-like growth factor				**x**		Id	
InR	Insulin-like peptide receptor				**x**		Id	
Chico	Insulin receptor substrate				**x**		Id	
PI3K	Phosphatidylinositol 3-kinase				**x**	**x**	Id	
Tor	Serine/threonine-protein kinase Tor				**x**	**x**	Id	
AKT	AKT serine/threonine protein kinase				**x**	**x**	Id	
Adcy	Adenylyl cyclase				**x**		Id	
OA	Octopamine receptors				**x**		Id	
Vg	Vitellogenin	**x**	**x**		**x**		Id	
Hex	Hexamerins				**x**		Id	

Among the many HSP families, HSP90 is one of the most abundant and universally expressed stress proteins. *Hsp90* gene is generally overexpressed during HSR (Brunt and Silver, 1991; Pratt and Toft, 2003; Sonoda et al., 2007), and has also been related to morphological evolution (Rutherford and Lindquist, 1998; Sollars et al., 2003; Specchia et al., 2010), reproduction, and brain development (Itoh et al., 1993; Izumoto and Herbert, 1993; Furay et al., 2006; Johnson and Brown, 2009). In *A. mellifera*, *Hsp90* is caste- and age-specifically expressed in adult bees (Aamodt, 2008), suggesting that *Hsp90* is expressed constitutively. In the genome of *A. mellifera*, two homologous cytoplasmic *Hsp90* genes are described, *Hsp83* and *Hsp90*, which are located in linkage group 1 (LG1) and LG7, respectively (Supplementary Table S2). These two genes produce two transcripts (A and B) by constitutive splicing (transcribed from the homolog in LG1 and LG7, respectively). Moreover, at least nine transcripts have been described from the LG7 gene by the AS under stress conditions (Xu et al., 2010).

HSP70s are a large family of ubiquitous molecular chaperones that protect cells from the damaging effects of many proteotoxic stresses [reviewed by Hendrick and Hartl (1993), Voos and Röttgers (2002), Richter et al. (2010), Walter and Ron (2011), Rosenzweig et al. (2019)]. *Hsp70 genes* are inducible, have no or relatively short introns and are preferentially translated, allowing HSP70 proteins to accumulate rapidly in response to adverse environmental stimuli (Gkouvitsas et al., 2009; Sørensen, 2010; Zhang and Denlinger, 2010). There are no relevant data about the genetic variability of *Hsp70* in *A. mellifera*, and three genes are annotated in its genome: *Hsp70Ab-like*, *Hsp70Cb*, and the mitochondrial *Trap-1* (Supplementary Table S2). Furthermore, the expression of *Hsp70 genes* is upregulated in response to many stressors (Hendrick and Hartl, 1993), and it seems to be a good indicator of colony stress in different *A. mellifera* subspecies (Alqarni et al., 2019; Morammazi and Shokrollahi, 2020).

Within the HSP70, there is another group of proteins called “cognate forms” or HSC70. The HSC70 protein family is structurally and functionally similar to HSP70, but its properties are different (Liu et al., 2012). *Hsc70 genes* are constitutive, and their corresponding proteins are involved in regulating the life cycle of various viruses, as it has been described in “Immunity” section. These genes contain more introns than those of *Hsp70*, and their number is conserved in vertebrates but it is variable in invertebrates (Chuang et al., 2007). In *A. mellifera* there are three annotated *Hsc* genes (Supplementary Table S2) and two of them have been linked to heat stress. HSC70-3 is a conserved endoplasmic reticulum chaperone (Pincus et al., 2010; Walter and Ron, 2011; Johnston et al., 2016; McKinstry et al., 2017) which is induced in honey bees under heat stress (i.e., 45 °C for 4 h) (McKinstry et al., 2017). Although viral infections alone induce *Hsc70-3* expression, heat shock alone did not always lead to overexpression. However, honey bees that were both virus-infected and heat-shocked displayed greater *Hsc70-3* expression (McMenamin et al., 2020). On the other hand, *Hsc70-4* is a core heat shock response gene that is induced by exposing honey bees to heat stress (i.e., 42 °C and 45 °C for 4 h) (Elekonich, 2009; Mahat et al., 2016; Solís et al., 2016; McKinstry et al., 2017). There is some heterogeneity in the expression of this gene, and genetic differences between subspecies may be one of the causes (McMenamin et al., 2020). The antiviral effect of both HSC70-3 and HSC70-4 proteins is due to the relationship between heat shock and RNAi machinery (discussed in the “Immunity” section below).

Chaperones of the HSP60 family are one of the most important components of the protein folding system in the mitochondrial matrix (Martin, 1997). HSP60, or chaperonins, forms a large homo-oligomeric protein complex with an inner cavity that provides a protected environment for the ATP-dependent folding of unfolded or newly synthesized single proteins or protein domains (Voos and Röttgers, 2002). HSP60 works with the cochaperone HSP10 (Voos and Röttgers, 2002), which is supposed to coordinate the behaviour of the single HSP60 monomers and regulate the ATPase cycle (Martin et al., 1993). In *A. m. ligustica*, highly expressed *Hsp60* has been described at 45°C (Alqarni et al., 2019), and *Hsp60* and *Hsp10* under heat and cold stress (Kim et al., 2019). In the genome of *A. mellifera,* there is one gene for each HSP60 and HSP10 protein (Supplementary Table S2).

The group of HSP40/J-domain-containing proteins are the largest class of HSP70 cofactors. They bind the nonnative protein and deliver it to HSP70. The J domains of these proteins interact with the ATPase domain of HSP70 and stimulate the hydrolysis of bound ATP (Kampinga and Craig, 2010). HSP40 is categorized into three subfamilies, namely, DnaJA, DnaJB, and DnaJC (Craig et al., 2006; Kampinga and Craig, 2010; Craig and Marszalek, 2017; Zhao et al., 2021). DnaJA1 regulates the expression of many antioxidant genes and heat shock genes (Figure 3), thereby improving the antioxidant ability of bees under heat stress (Li et al., 2020a). It is important to note that the expression levels of *DnaJA1*, *DnaJB12* and *DnaJC8* are upregulated under UV radiation, cold, and pesticide treatment in *A. cerana cerana*, and their silencing attenuates the resistance of this subspecies to λ-cyhalothrin stress (Li et al., 2018a). Within the genome of *A. mellifera* there are 18 genes of the DnaJ family (Protein lethal (2) essential for life-like) (Supplementary Table S2).

Finally, small HSPs (sHSPs) are a wide family of proteins involved in HSR but also have protective functions under different stresses, such as cold, drought, oxidation, hypertonic stress, UV, heavy metals, and even stress by high population density (Dasgupta et al., 1992; Wang et al., 2007; Waters et al., 2008; Shih et al., 2021). It is important to note that some sHSPs also have a chaperone function in development (Sun and MacRae, 2005). Thus, their corresponding genes appear to be a family of both constitutive and inducible genes. Unfortunately, at present, there is no formal classification of this gene family in *A. mellifera* according to the pattern of gene expression. Interspecific genetic variability has been described in the genes coding for small HSPs. Insect sHSPs are generally species-specific, suggesting that functions of most sHSPs may have diverged across species. This variability likely reflects the role of sHSPs in the adaptation of insects to diverse ecological niches (Li et al., 2009). The C-terminal sequences of these proteins harbours the conserved α-crystallin domain, while the N-terminal remain variable. This indicates that the conserved C-terminal has a significant part in sustaining the chaperone and other functions, whereas the N-terminal may be associated with diverse expressions, functions, and evolutionary patterns within sHSPs (Li et al., 2009). On the other hand, most sHSP genes that are located on a single chromosome are usually arranged in tandem. This arrangement may enable organisms to rapidly respond to changing environmental conditions due to regulatory advantages. Tandem *sHsp* genes may be a better way for insects to regulate gene expressions in diverse environments (Li et al., 2009). In the *A. mellifera* genome there are eight *sHsp genes* annotated as *protein lethal (2) essential for life* (Supplementary Table S2).

### 4.2 Other genes related to heat stress

#### 4.2.1 Nuclear Factor-Y

The Nuclear Factor Y (NF-Y), also known as Heme Activator Protein (HAP) or CCAAT-Binding Factor (CBF), consists of three distinct subunits (NF-YA, NF-YB, and NF-YC) which are found in almost all organisms (Li et al., 2018b; Myers and Holt, 2018). Each animal NF-Y subunit is typically encoded by only one gene, the product of which can undergo different post-translational modifications and have various splicing forms (Li et al., 1992; Mantovani, 1999; Fujikake et al., 2005). In *Drosophila melanogaster*, NF-Y is essential for the growth and development of the thorax, eye, and R7 photoreceptors, as it regulates multiple signalling pathways, such as the extracellular signal-regulated kinases (ERK) and the c-Jun N-terminal kinases (JNK) pathways (Yamaguchi et al., 2017; Li et al., 2018b). In bees, the expression levels of *NF-YA, NF-YB,* and *NF-YC* are induced by long- and short-term heat stress in *A. c. cerana* and *A. mellifera* (Zhao et al., 2021). In *A. c. cerana*, the knockdown of *NF-YB* and *NF-YC* decreases the antioxidant capacity and increases the oxidative damage caused by heat (Figure 3). Upregulation of *NF-Y* may increase the heat resistance of bees under different heat stress conditions by reducing oxidative damage and enhancing antioxidant ability (Li et al., 2020b). The expression of *NF-YA, NF-YB*, and *NF-YC* in *A. mellifera* is not only induced by heat but also by several stress conditions (Table 1), including cold and UV light (Li et al., 2020b). There are four *NF-Y* genes annotated in the genome of *A. mellifera* (Supplementary Table S3).

#### 4.2.2 Zinc finger proteins

Zinc finger proteins (ZFP) are among the most abundant proteins in eukaryotic genomes. Their functions are diverse and include DNA recognition, RNA packaging, transcriptional activation, regulation of apoptosis, protein folding and assembly, and lipid binding (Laity et al., 2001). The expression of some genes encoding ZFP has been linked to heat stress (Droll et al., 2013; Liu et al., 2015) (Figure 3), cold, pesticides, and UV exposure in *A. cerana*, suggesting an important role of these proteins in resistance to a variety of environmental stressors (Guo et al., 2021) (Table 1). In *A. mellifera*, five *ZFP* genes (*ZFP271, ZFP37, ZFP239, ZFP776,* and *ZFP93*) have been described as upregulated under high-temperature exposure, while two (*ZFP62* and *ZFP628*) have been described as downregulated (Ma et al., 2019). There are several *ZFP genes* annotated along the genome of *A. mellifera*, so here we summarize the seven already cited (Supplementary Table S3). There are four loci annotated for *ZFP271*, two for *ZFP37,* two for *ZFP239,* and only one for *zinc finger protein 776-like*. The *ZFP93* gene does not appear in the genome assembly of *A. mellifera*, but it encodes a ZFP of the KRAB (Kruppel-associated box) subfamily (Bellefroid et al., 1993), and there is a *kruppel homolog 1* (*Kr-h1*) gene in *A. mellifera*. Finally, the two genes described downregulated are *ZFP62* and *ZFP628-like*. Regarding AS, different isoforms from *ZFP genes* have been predicted and annotated in GenBank.

#### 4.2.3 Serine proteases (SPs)

Serine proteases (SP) are endo- and exopeptidases involved in insect immunity and antioxidant systems (Ashida and Brey, 1997), as well as the heat stress response in different bee species (Table 1). For example, in *A. cerana,* the gene enconding for the Clip-domain serine protease1 (*AccSp1*) is upregulated by temperature (4, 24 °C and 44 °C), H_2_O_2_, heavy metals, UV-light, and pesticides, thus linking SPs to its defence against abiotic stresses (Gao et al., 2019). There is a *Sp1* gene annotated in the genome of *A. mellifera* (Supplementary Table S3). Regarding biotic stressors, SPs are directly involved in immunity, participating in the prophenoloxidase (PPO) activation pathway, RNA interference, and SP proteolytic cascade in the Toll signalling, as described below in the “Immunity” section. Compared with *D. melanogaster* and *Anopheles gambiae*, *A. mellifera* has much smaller gene families of SP, SPH, serpin (Serine Protease Inhibitors), PPO and other immune proteins (Evans et al., 2006). A search of the *A. mellifera* genome yielded 57 sequences with significant similarity to the S1 protease family: 44 SP and 13 SPH genes (Zou et al., 2006). SPHs are similar in sequence to S1 proteases but lack one or more of the catalytic residues in SPs. In addition, seven annotated genes in the honey bee genome encode five serpins (serpin 1–5) and two serpin-like proteins (Zou et al., 2006). SP inhibitors of the serpin superfamily are present in insect haemolymph to remove excess proteases and maintain homeostasis (Kanost, 1999). Finally, genes of SP putative substrates prophenoloxidase (PPO) and spätzle are described in the *A. mellifera* genome (Zou et al., 2006) (Supplementary Table S3).

#### 4.2.4 Antioxidant (AO) enzymes

Alterations in the oxidation states of intracellular metabolites and enzymes have historically been considered negative stressors, requiring strictly defensive responses. Cellular growth and survival require the coupling of electron-transfer reactions to the generation of ATP. These reactions depend on key cellular electron carriers and the stability of protein residues and cofactors. Redox enzymes are notoriously nonspecific, transferring electrons to any suitable acceptor they encounter. These molecular mechanisms represent a constant cellular stress balanced and maintained by redox homeostasis (Sporer et al., 2017). There are both primary and secondary antioxidant enzymes, which act directly or indirectly on reactive oxygen species (ROS) molecules. The first line of defence against ROS attack is provided by three different kinds of primary antioxidant enzymes, which act directly on ROS: superoxide dismutase, catalase and peroxidases. Superoxide dismutase (SOD) rearranges superoxide to oxygen and hydrogen peroxide, catalase prevents free hydroxyl radical formation by breaking down hydrogen peroxide into oxygen and water, and peroxidases (POD) catalyse an analogous reaction in which hydrogen peroxide is reduced to water by a reductant that acts as an electron donor, normally reduced thioredoxin (TRX) or glutathione (GSH). In addition, insects have three families of genes that encode antioxidant enzymes that act as peroxidases: TPXs, also known as peroxiredoxins (Radyuk et al., 2001), phospholipid-hydroperoxide GPX homologs with thioredoxin peroxidase activity (GTPX) (Missirlis et al., 2003), and glutathione S-transferases (GSTs) (Tang and Tu, 1994; Toba and Aigaki, 2000). Secondary antioxidant enzymes that act indirectly on ROS include thioredoxin (TrxR) and methionine sulphoxide reductases (MsrA and MsrB), which are involved in protein reparation by catalysing the TRX-dependent reduction of methionine sulphoxide to methionine (Moskovitz et al., 1996; Kumar et al., 2002).

In *A. mellifera* genome, 38 antioxidant genes were identified. In general, antioxidant genes encode small proteins with less than 250 amino acids, and most of them possess at least one intron (Corona and Robinson, 2006). Alternative splicing has been described as a common mechanism in the RNA processing of these genes, suggesting that the resulting different isoforms may play a role in stress resistance. About genetic variability, 52 and 29 alleles are described for *SOD1* and *SOD2* in *D. melanogaster*, as well as 34 alleles for catalase (FlyBase, 2025). On the other hand, there is a relationship between redox enzymes and HSR (Figure 3). High temperatures exponentially increase metabolic rates, which means higher oxygen consumption and therefore higher production of ROS and more oxidative damage to organisms (Finkel and Holbrook, 2000; Gillooly et al., 2001; Belhadj Slimen et al., 2014; González-Tokman et al., 2020). Under long-term stress, the expression of additional *Hsp* and other gene family members is upregulated, which in turn scavenges ROS, enhances the antioxidant defence system of bees, and increases their survival rate [reviewed by Zhao et al. (2021)]. As mentioned in the “Heat Shock Proteins” subsection, DnaJA1 (HSP40) can regulate the expression of many antioxidant genes, thereby improving the antioxidant ability of bees under heat stress (Li et al., 2020b). It is important to note that oxidative stress response mechanisms are activated by many types of factors, including not only heat stress but also nutritional stress, pesticides, pathogens, UV radiation, among others (Table 1). On the other hand, immunity molecular pathways can influence ROS levels by increasing the expression of antioxidant proteins (Zhang et al., 2016), as described in the “Immunity” section. Genes encoding antioxidative enzymes in *A. mellifera* are noted in Supplementary Material (Supplementary Table S3).

#### 4.2.5 Acetylcholinesterase

Acetylcholinesterase (AChE) is a serine hydrolase that controls synaptic and neurohumoral cholinergic activity by hydrolyzing the neurotransmitter acetylcholine into acetic acid and choline [reviewed by Silman and Sussman (2008)]. *A. mellifera* has two genes, *Ace1* and *Ace2*, that encode AChE1 and AChE2 proteins, respectively (Supplementary Table S3). In bee species belonging to *Bombus* and *Apis*, AChE2 acts as the synaptic enzyme, while AChE1 shows little catalytic activity. This suggests that AChE1 may have become specialized to play non-synaptic functions (Kim and Lee, 2013). In *A. mellifera*, the expression of *Ace1* changes depending on the rearing stage of the colony (Kim et al., 2017), and it appears to be connected to HSR (Kim et al., 2019). In addition, the genetic variability of *Ace1* linked to pesticide resistance has been described in several insect species (Nabeshima et al., 2003; 2004; Weill et al., 2004; Baek et al., 2005; Oh et al., 2006; Alon et al., 2008; Kozaki et al., 2008; Jiang et al., 2009; Ramphul et al., 2009; Wu et al., 2010). AChE is the main target of organophosphorus (OP) and carbamate (CB) insecticides, to which *A. mellifera* displays unique sensitivity profiles (Hardstone and Scott, 2010), perhaps because AChE2 is its main synaptic AChE (Kim and Lee, 2013).

#### 4.2.6 Corticotropin-releasing hormone-binding protein (CRH-BP)

Throughout evolution, highly conserved signalling molecules have been utilized to integrate stress responses, highlighting their important roles in survival. The corticotropin-releasing hormone (CRH), also known as corticotropin-releasing factor (CRF), is a neuroendocrine peptide that regulates various physiological responses to stresses (Seasholtz et al., 2002). It is considered to act as a neurotransmitter, coordinating various autonomic, hormonal, and behavioural responses to stresses, and may be involved in developmental processes (Muglia et al., 1995; Cortright et al., 1997; Majzoub, 2006). The high homology of the CRH binding protein (CRH-BP) in honey bees to that in humans suggests that its function(s) has also been evolutionarily conserved (Ketchesin et al., 2017). In *A. mellifera*, CRH-BP is a 322-amino-acid soluble protein structurally unrelated to the CRH receptors. The *CRH-BP gene* (Supplementary Tables S3 and S5) is well-conserved and identifiable in insects. Although very few studies have focused on CRH-BP and stress in invertebrates, the Chinese honey bee *A. cerana cerana* subjected to UV light, heat, or cold exhibited increased CRH-BP mRNA in the head in a time-dependent manner (Liu et al., 2011). These studies highlight the upregulation of invertebrate CRH-BP mRNA in response to stress (Ketchesin et al., 2017). Therefore, CHR-BP seems to be a promising protein candidate as a potential element involved in the stress response, and its action would be worth investigating in future studies (Even et al., 2012).

## 5 Relative humidity

While temperature has been the main focus of climate change studies, changes in precipitation patterns are also key, not only because of water availability but also because of fluctuations in relative humidity (RH) (IPCC-Intergovernmental Panel on Climate Change, 2007). In *A. mellifera* colonies, a suitable RH of up to 75% is required for egg hatching (Ellis et al., 2008), and changes in RH can significantly affect larval development during incubation (Human et al., 2006) [reviewed by Abou-Shaara et al. (2017)]. In fact, when RH falls below 50%, there is a significant reduction in the number of normally hatched eggs (Li et al., 2016). Comparative studies of *A. mellifera* subspecies revealed that *A.m. jemenitica* eggs exhibit higher hatching rates than those of *A.m. carnica* at 50%–75% RH, while no eggs from either subspecies hatched at 30% RH (Al-Ghamdi et al., 2014).

The bees can regulate humidity within the colony by evaporating water from nectar and regurgitating liquid droplets, helping to restore favourable RH conditions while also aiding in thermoregulation (Heinrich, 1980; Human et al., 2006; Kovac et al., 2007). As for external conditions, no clear direct impact of RH in honey bees, including foraging activity, has been reported (Joshi and Joshi, 2010).

High humidity alone appears to have a limited impact on bees compared to high temperatures (Ma et al., 2019). However, rather than acting as an isolated stressor, humidity most relevant effect likely occurs in combination with temperature. These two variables are closely interconnected and, when combined, act as a new independent stressor. For example, low RH levels combined with high temperature can exacerbate heat stress, whereas high RH can reduce its severity. The highest rate of body water loss in *A. mellifera* occurs at 35 °C/0% RH, and the lowest occurs at 25 °C/75% RH and 30 °C/100% (Atmowidjojo et al., 1997). Studies on *A. m. jemenitica* and *A. m. carnica* showed that body water loss rates increased with higher temperature and lower RH, while higher RH generally improved workers’ survival (Abou-Shaara et al., 2012). In *A. mellifera* and *A. cerana*, high RH was shown to have a protective effect on bee survival under high temperatures, which may be due to the inhibition of body water loss (Li et al., 2019). Therefore, although the effect of RH on bees was previously considered negligible (Joshi and Joshi, 2010), it appears to become an important stress factor when it drops below 50% in warm environments, conditions increasingly common in arid regions under a climate change scenario.

The protective effect of high RH under high temperatures is also evident at the molecular level. Ma et al. (2019) demonstrated this in their study on *A. mellifer*a when examining its responses to various combinations of temperatures and RH. The authors reported 434 differentially expressed genes (DEGs) under high-temperature treatments, 86 under high-humidity treatments, and 266 in combined high-temperature and humidity treatments. This suggests that high humidity reduces the expression of heat response genes by nearly half in a heat-stress environment. However, when focusing solely on molecular responses to humidity stress under optimal temperature conditions, the expression of genes such as *Pla2* (phospholipase A2) and *Afp* (antifreeze protein) increases with increasing humidity (Table 1), starting at RH levels above 50% (Ma et al., 2019). *Pla2* encodes a cell membrane enzyme (PLA2) that cleaves fatty acids. When its activity increases, the hydrolysis reaction is enhanced, producing a variety of fatty acids, disturbing cell membrane metabolism and nerve signal transmission (Mahalka et al., 2011). It is important to note that the PLA2 protein is involved in immunity through the arachidonic acid release in the eicosanoid pathway (McMenamin et al., 2018). There are 10 genes in the genome of *A. mellifera* referred to *Pla2* (Supplementary Table S4). *Afp* encodes AFP, a proteinaceous compound with enhanced antifreeze properties, enabling it to bind to small ice crystals and inhibiting their growth and recrystallization. This mechanism helps minimize the damage caused by frozen water/ice to living organisms (Jia and Davies, 2002). There is one gene in the genome of *A. mellifera* referred to *Afp* (Supplementary Table S4).

## 6 Ultraviolet exposure

Climate change has been linked to stratospheric ozone depletion, which increases the amount of ultraviolet-B (UV-B) radiation that reaches the Earth’s surface (IPCC-Intergovernmental Panel on Climate Change, 2007). This affects how organisms and ecosystems respond to it (Bernhard et al., 2020). UV radiation is well-known for its detrimental effect on organisms. Yet, it is the universal source of non-ionizing radiation and is essential for life and its development on Earth. According to CIE (Commission Internationale de l’Eclairage, International Commission on Illumination), UV radiation can be divided into three ranges: UVA (320–400 nm), UVB (280–320 nm), and UVC (200–280 nm). Earth’s ozone layer blocks the majority of UVC, and a significant portion of UV-B, so the light near the Earth’s surface is enriched by UV-A, which has also been linked to oxidative types of mutation due to oxidative stress following irradiation [reviewed by Cockell and Knowland (1999)].

UV promotes photochemical reactions of ROS formation. In fact, one of the most susceptible biological targets of UV radiation is the DNA. When UV radiation enters a cell, it is absorbed by the aromatic rings of nucleotides and amino acids, leading to DNA and protein damage, respectively [reviewed by Cockell and Knowland (1999)]. However, it is important to note that repair mechanisms are not 100% efficient, so the lower the exposure to UV-radiation, the lower the damage and the greater the benefit to the organism [reviewed by Cockell and Knowland (1999)]. Hence, UV protection is key, particularly in a scenario of climate change with a prospective increase of UV radiation exposure.

Cells use a wide array of biological macromolecules to protect themselves against ROS by quenching oxygen free radicals. In insects, melanins are good examples of such macromolecules. Melanins can be classified into two groups: brown to black pigments termed eumelanin, and alkali-soluble yellow to reddish-brown pigments termed pheomelanin (Ito and Wakamatsu, 2008). They enhance the protective properties of the cuticle, acting as important barriers against environmental stressors such as UV radiation (Charlier et al., 2020). Melanins are produced by the enzymatic oxidation of tyrosine by tyrosinase followed by the conversion of dopa to 5,6-dihydroxy-indole. This is a phenolic and indolic compound and the basic building block of the eumelanin polymeric structure. This structure acts as the UV-absorbing chromophore [reviewed by Cockell and Knowland (1999)]. Recently, Charlier et al. (2020) used the electron paramagnetic resonance imaging (EPRI) technique to detect melanin in *A. mellifera*. They identified free radicals almost exclusively in the cuticle of the bee periphery of *A. mellifera* consistent with a eumelanin signal. This finding suggests that melanin–chitin complexes in the honey bee cuticle play a key role in UV defence (Charlier et al., 2020). Interestingly, these authors detected the presence of other free radicals in the centre of the honey bee head, suggesting the possible presence of neuromelanin in its brain, as in *Drosophila* (Barek et al., 2018).

At the molecular level, melanization is an immunological process that results from the combination of humoral and cellular processes that occur during encapsulation and healing to cope with non-pathogen-mediated and pathogen-mediated injuries. In the honey bee, and insects in general, melanization acts as an important cellular defence mechanism responsible for eliminating a large number of bacterial cells, parasites and xenobiotics (Eleftherianos et al., 2009; McMenamin et al., 2018). Simultaneously with the formation of melanin and its polymerization together with other proteins to encapsulate the invading agent, reactive oxygen and nitrogen intermediates are released, which collaborate with the destruction of the agent (Negri et al., 2016). Melanization is mediated by a humoral protein, prophenoloxidase (PPO) (González-Santoyo and Córdoba-Aguilar, 2012; Negri et al., 2019) and *A. mellifera* possesses only one *PPO* gene (Supplementary Table S5).

Several markers involved in temperature stress (see “Temperature” section) are also involved in UV stress (Table 1). For example, in *A*. *cerana*, the expression of DnaJA1, DnaJB12 and DnaJC8 are upregulated under exposure to both climatic stressors (Li et al., 2018a). Furthermore, as for temperature, sHSPs can develop the protection function under UV stress conditions not only in honey bees but also in other animals and even plants (Dasgupta et al., 1992; Waters et al., 2008). Although very few studies have focused on CRH-BP response to stress in invertebrates, it has been shown that *A*. *cerana cerana* subjected to UV radiation exhibited increased CRH-BP mRNA in the head in a time-dependent manner (Liu et al., 2011). On the other hand, it is worth noting that UV promotes photochemical reactions leading to ROS formation, so antioxidant enzymes are also involved in UV stress. Finally, the serine protease *AccSp1* gene, which is directly involved in ROS metabolism, seems to play different roles in resistance to UV radiation in *A. cerana* (Gao et al., 2019).

## 7 Nutrition

Although the interactions between plants and their insect pollinators are often the result of a long history of co-evolution, climate change can quickly disrupt the timing of their life cycles by altering their phenology and distribution (Hughes, 2000; Scaven and Rafferty, 2013; Gérard et al., 2020). Changes in plant physiology under realistic climate change scenarios may alter flowering patterns and the duration and intensity of blooming (Scaven and Rafferty, 2013; Inouye, 2020). This disruption could lead to food shortages for pollinators and, in some cases, even contribute to their extinction (Memmott et al., 2007; Dalton et al., 2023).

The abundance and diversity of floral resources play a crucial role in honey bee health, as they significantly influence immune response (Alaux et al., 2010; Di Pasquale et al., 2013; Martelli et al., 2022; Corona et al., 2023). The survival of bee colonies relies heavily on the availability of pollen, which provides proteins, lipids, and micronutrients, and nectar, which supplies carbohydrates (Brodschneider and Crailsheim, 2010). In temperate climates, bees typically experience nutritional stress as winter approaches due to the diminishing availability of food resources (Mattila and Otis, 2007; Knoll et al., 2024). This issue has gained particular attention because many bees face low dietary diversity due to climate change (Goulson et al., 2015).

**FIGURE 4:**
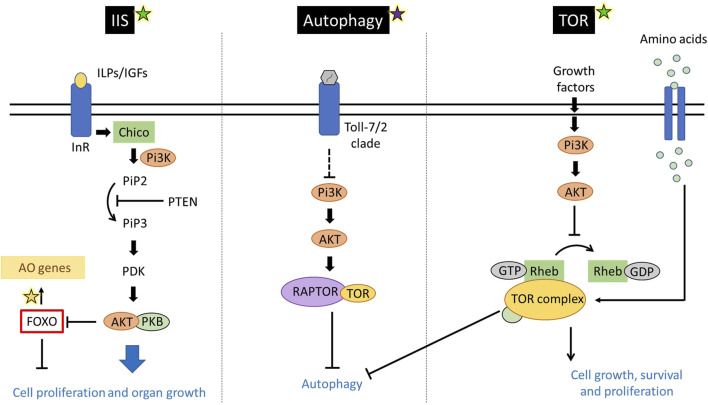
Nutritional molecular pathways connected to immunity. In the insulin/insulin-like signalling (IIS) pathway, insulin-like peptides (ILPs) and insulin-like growth factors (IGFs) bind to the insulin receptor and CHICO receptor substrate. Phosfatidylinositol-3-OH kinase (Pi3K) activates the phosphatidylinositol (PiP2 to PiP3) reaction ultimately promoting cell differentiation, and organ growth and thereby an increase in lifespan (Pan and Finkel, 2017). The AGC family kinase AKT inhibits the Forkhead box O subfamily of gene (FOXO). The FOXO proteins play an antioxidant role and regulate the autophagy process. TOR pathway is stimulated by growth factors and free amino acids but inhibited by hypoxia and ATP depletion (Abraham, 2009). TOR is a serine/threonine protein kinase belonging to the phosphoinositide 3-kinase-related family, which is highly conserved among eukaryotes (Pan and Finkel, 2017). Pi3K plays a role in the TOR pathway when it is stimulated by growth factors, activating AKT. Yellow, purple and green stars indicate relationship with antioxidants, immune pathways, and nutrition, respectively (Abraham, 2009; Emlen et al., 2012; Koyama et al., 2013; McMenamin et al., 2018)**.**

At the molecular level, bees that consume a protein-rich diet exhibit signs of anabolism, lipid metabolism, and an increased expression of genes encoding nursing-related proteins, such as vitellogenin and major royal jelly protein 1 (Bitondi and Simoes, 1996; Corona et al., 2007; 2023; Ament et al., 2008; Azzouz-Olden et al., 2018). In contrast, bees subjected to suboptimal nutrition, typically characterized by the intake of diets lacking proteins, display a reduction in nitrogen and lipid metabolism, reduction in antioxidant response, and different gene expression patterns (Martelli et al., 2022). These include altered expression in cuticle maturation genes, the over-expression of genes involved in the modulation of circadian rhythm and other genes related to the foraging behavioral state, such as those involved in the insulin/insulin-like growth signaling (IIS), juvenile hormone (JH) and Target of Rapamycin (TOR) (Figure 4) (Supplementary Table S6) (Corona et al., 2007; 2023; Ament et al., 2008; Azzouz-Olden et al., 2018; Martelli et al., 2022). These nutrient-sensing pathways include the Forkhead box O subfamily of gene (FOXO) [reviewed by Murtaza et al. (2017)], a transcription factor involved in multiple biological processes including the regulation of aging, nutrient levels response, and stress response (Hwangbo et al., 2004; Semaniuk et al., 2021a; Semaniuk et al., 2021b). FOXO proteins are known to regulate translation of environment-induced stimuli into gene expression and their antioxidant role [reviewed by Murtaza et al. (2017)]. In *A. mellifera* genome there is only one *FOXO* gene (Supplementary Table S6).

The connection between honey bee nutrition and behavioral development is further highlighted by the fact that nutritional stress is associated to precocious foraging (Fewell and Winston, 1992; Schulz et al., 1998), changes in colony demographics, and, ultimately, colony collapse (Perry et al., 2015). Research indicates that similarly to nutritional stress, thermal stress reduces the expression levels of nurse-associated genes (*vg* and *mrjp1)* and increases the expression of foraging-associated genes (e.g., juvenile hormone esterase) (Bordier et al., 2017; Corona et al., 2023) suggesting that thermal stress can induce the physiological changes linked to precocious foraging, potentially affecting the overall fitness of the colony. In support of this idea, it has been shown that bees raised under high temperatures exhibited an increased probability of dancing and foraging earlier in life (Becher et al., 2009).

### 7.1 Phenolamines

Unlike vertebrates, honey bees do not synthesize the catecholamines norepinephrine and epinephrine but use instead the phenolamines tyramine (TA) and octopamine (OA) to perform similar physiological functions (Roeder, 2020). Both biogenic amines affect the locomotor behaviour of adult worker honey bees (Fussnecker et al., 2006). Additionally, OA plays an important role in associative learning and memory (Menzel et al., 1996; Blenau and Baumann, 2001; Roeder et al., 2003) by mediating and modulating the reward in appetitive learning (Hammer and Menzel, 1995; Scheiner et al., 2006; Kim et al., 2013). The activation of receptors of tyramine and octopamine are closely related to adenylyl cyclase activity, which can be activated or inhibited in order of the receptor type or subtype (Blenau et al., 2000; Grohmann et al., 2003; Mustard et al., 2005; Beggs et al., 2011; Balfanz et al., 2014; Reim et al., 2017). Adenylyl cyclase (or adenylate cyclase) catalyzes the conversion of ATP to 3′,5′-cyclic AMP. Genes encoding adenylyl cyclase (Ac76E) and the octopamine receptor (OA) have been described as “starvation genes” in underfed honey bees (Azzouz-Olden et al., 2018). Six adenylyl cyclase genes and four octopamine receptor genes have been annotated in the *A. mellifera* genome (Supplementary Table S6).

### 7.2 Storage proteins

Insect storage proteins are an important source of amino acids, particularly during metamorphosis. In honey bees, the most known storage protein is the lipoprotein Vitellogenin (Vg), which possesses multiple functions such as royal jelly production (Amdam et al., 2003), promotion of longevity (Amdam and Omholt, 2002; Seehuus et al., 2006; Corona et al., 2007), and immunity (Amdam et al., 2004). Vg has also been proposed as a plausible candidate for a stress marker together with the juvenile hormone (JH) which is also considered to be related to stress responses. Both are involved in oxidative stress (Seehuus et al., 2006; Corona et al., 2007) and heat stress in *A. mellifera* (Bordier et al., 2017). There is only one *Vg* gene in *A. mellifera* genome (Supplementary Table S6), but three *Vg-like* genes have also been described with different evolutionary patterns and functions (Salmela et al., 2016).

Other important storage proteins in honey bees are the hexamerins. They are the most abundant proteins in larval haemolymph and essentially participate in the dynamics of amino acid storage and exploitation, which occurs during insect development (Telfer et al., 1991). There are four hexamerin genes in the honey bee genome corresponding to subunits 70A, 70B, 70C and 110 (Supplementary Table S6). The *hex70a*, *hex70b* and *hex70c* genes are arranged in tandem in chromosome 8, whereas *hex110* is dispersed in chromosome 11, exhibiting unusual features throughout its sequence (Martins et al., 2010). The JH exerts a strong and positive influence on the expression of *hex70b* and *hex70c*, while its effect on the expression of *hex70a* and *hex110* is comparatively weaker (Martins et al., 2010). As for nutritional effects, similar to Vg, hexamerins expression increases when honey bees are fed pollen (DeGrandi-Hoffman et al., 2021), thus being reliable indicators of good nutritional status.

## 8 Other cellular mechanisms involved in stress resistance

In addition to the pathways that are directly or indirectly involved in stress management, cells have several general mechanisms that are activated in stressful situations, such as changes in RNA processing and epigenetic factors. They all act independently of the organism’s genetic background and directly influence its phenotypic plasticity (Figure 1), and, therefore, its resistance and adaptability to new environments. This section reviews the major ones in *A. mellifera* and their role in stress.

### 8.1 Alternative splicing

**FIGURE 5:**
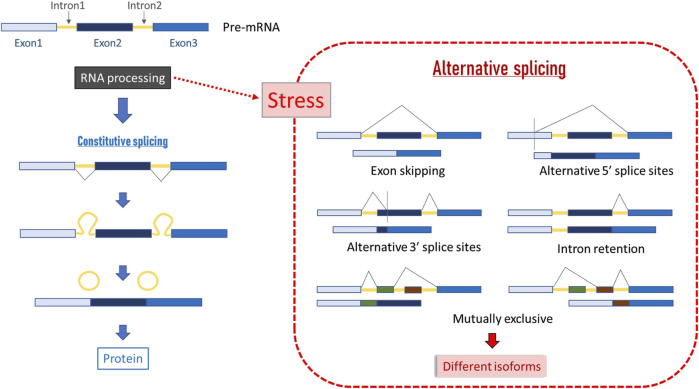
Scheme of constitutive and alternative splicing. In constitutive splicing, total coding DNA is retained in the final mRNA product and translated into a protein, and all introns are fully spliced during posttranscriptional RNA processing. In alternative splicing, introns can be spliced in more than one way, resulting in different sets of exons in the mature mRNA, and hence, in different series of related proteins called isoforms (Ast, 2004).

Modulation of RNA processing is involved in stress tolerance in insects (Fujikake et al., 2005; Ding et al., 2014; Ruan et al., 2015; Pai et al., 2017). In RNA processing, the splicing machinery (spliceosome) recognises exons with high accuracy, removes the introns from the pre-mRNA molecule, and ligates the exons to form a mature mRNA. This process is known as “constitutive splicing.” On the other hand, alternative splicing (AS) is the process by which the exons of primary transcripts (pre-mRNAs) can be spliced in different arrangements to produce structurally and functionally distinct mRNA and protein variants, or isoforms (Figure 5). AS is involved in many physiological processes, including the response to biotic and abiotic stresses [reviewed by Biamonti and Caceres (2009)]. The production of proteins with diverse domain rearrangements from the same gene is the main AS mechanism for pathogen-resistance genes. These mRNA variants have been identified for many genes, particularly those involved in the regulation of stress responses, such as protein kinases, transcription factors, splicing regulators and pathogen-resistance genes (Mastrangelo et al., 2012). This occurs according to physiological needs and environmental stimuli, and often represents a primary source of phenotypic diversity within the proteome of eukaryotic cells (Ast, 2004; Blencowe, 2006; Neves-da-Rocha et al., 2019). Therefore, alternative-splicing isoforms produced by stress-related genes could be used as proxies of stress.

### 8.2 Epigenetics

Epigenetic phenomena include all processes by which the expression of a gene can be altered (overexpressed or silenced), resulting in a phenotypic change while the genotype remains unchanged. The best-known epigenetic phenomena are the methylation of nucleotides (i.e., fixation of a methyl radical on a nucleotide) and changes in the configuration of histones. Both mechanisms are the major regulators of gene expression in all organisms (Glastad et al., 2019) and act as a major source of phenotypic plasticity (Berger et al., 2009). The genome of *A. mellifera* has been described as structured with respect to plasticity, with genes related to stress response being organized into clusters that are marked by histone modifications (Duncan et al., 2020). Epigenetic mechanisms can be triggered by environmental factors such as heavy metals or persistent organic pollutants, which can modulate epigenetic marks such as acetylation or methylation (Foret et al., 2009; Collotta et al., 2013). With regard to climate change, DNA methylation and histone/chromatin modifications have been linked to thermal stress responses and facilitate transgenerational epigenetic inheritance of thermal adaptation [reviewed by McCaw et al. (2020), de Carvalho (2023)]. These modifications also enable populations to adapt to local and global climate gradients.

#### 8.2.1 DNA methylation

**FIGURE 6:**
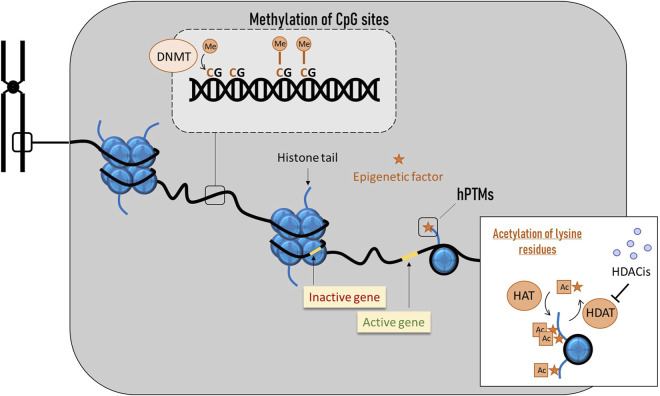
Main epigenetic phenomena in honey bees. DNA methylation occurs by the addition of a methyl group to the fifth carbon of cytosines, mostly in CG dinucleotides (CpG). Histone modifications involve the disruption of histone-DNA interactions, causing nucleosomes to unwind and genes to become accessible to the transcriptional machinery, allowing subsequent gene activation.

DNA methylation is a covalent modification that occurs by the addition of a methyl group to the fifth carbon of cytosines, mostly in CG dinucleotides (CpG) (Klose and Bird, 2006; Suzuki and Bird, 2008; Zemach et al., 2010; Moore et al., 2013) (Figure 6), although adenine methylation can also occur (Ratel et al., 2006). Moreover, DNA is not the only nucleic acid that can be modified; RNA can also undergo modifications that impact gene expression post-transcriptionally (Sieber et al., 2021), creating a new layer of dynamic gene regulation [reviewed by Kan et al. (2022)]. Recent studies suggest that the epitranscriptome of honeybees may play a role in stress responses (Bataglia et al., 2021; 2022).

In contrast to the heavily methylated genomes of mammals, the invertebrate genomes are sparsely methylated in a “mosaic” fashion, with most methylated CpG dinucleotides found across gene bodies (Wedd and Maleszka, 2016). The gene body methylation is frequently associated with active transcription (Wedd et al., 2022), and it seems to be an important feature of caste determination in social insects (Sieber et al., 2021). Some studies on the honey bee have shown strong links between gene body methylation and AS (Wedd et al., 2016). In addition, the existence of non-CpG methylation events in honey bee introns, potentially playing a role in the regulation of AS, has been also proposed (Cingolani et al., 2013).

DNA methylation is controlled by DNA methyltransferases (DNMTs). DNMT1 is responsible for maintaining methylation states across cell divisions, whereas DNMT3 is involved in *de novo* methylation (Klose and Bird, 2006), although these functions can overlap (Jeltsch and Jurkowska, 2014; Karemaker and Baubec, 2020). DNA methylation can be reversible, in particular through the action of the ten/eleven translocation (TET) family enzymes (Kohli and Zhang, 2013). Regarding maintained methylation states, allele-specific methylation is known to occur in honey bees (Remnant et al., 2016; Wedd et al., 2016). Interestingly, honey bee males seem to harbour individual-specific DNA methylation patterns in their semen and these patterns are often associated with genotypic variation. It means that genes that are variable at the epigenetic level appear to be more likely to be variable at the genetic level. This sequence polymorphism could be an important determinant of the DNA methylation state at many loci in honey bees, contributing both to the individual-specificity of epigenetic marks and to their retention across generations (Yagound et al., 2019). While there is evidence that gene expression patterns are sometimes heritable, additional experimental analyses are required to conclusively demonstrate that DNA methylation is the epigenetic cause of such heritable effect (Glastad et al., 2019).

#### 8.2.2 Histone post-translational modifications (hPTMs)

Eukaryotic DNA is packaged in basic and repeating structural units (nucleosomes), where a segment of DNA wound around the histone cores (Figure 6). These histone cores are composed of several subunits (H2A, H2B, H3 and H4), and each one contains amino acid tails that are sites of post-translational regulation (Spotswood and Turner, 2002). Some modifications disrupt histone-DNA interactions, causing nucleosomes to unwind. In this open chromatin conformation, DNA becomes accessible to the transcriptional machinery, enabling subsequent gene activation. In contrast, modifications that reinforce histone-DNA interactions create a very compact chromatin where the transcriptional machinery cannot access the DNA, resulting in gene silencing.

Histone post-translational modifications (hPTMs) consist of a diverse set of epigenetic signals that can alter transcription either by the addition of a chemical group to a histone protein or by specific protein binding to histone tails (Glastad et al., 2019). Regarding the former mechanism, lysine acetylation is perhaps the most studied modification, as it was one of the first discovered to influence transcriptional regulation. Acetylation of lysine residues results in neutralization of histone charge, weakening the nucleosome structure and making DNA accessible for transcriptional factors binding, significantly increasing gene expression (Roth et al., 2001). Acetyl groups are added to lysine residues of histones H3 and H4 by histone acetyltransferases (HAT) and removed by deacetylases (HDAC). The action of these two types of enzymes results in opposite gene expression outcomes (Bernstein et al., 2007). Diverse compounds can inhibit deacetylases, and they are known as HDAC inhibitors (HDACis). They act by triggering histone tail acetylation and play an important role in epigenetic gene regulation (Marks et al., 2003). In insects, HDACis can accelerate growth, extend longevity, and help overcome injuries (Zhao et al., 2005; Mukherjee et al., 2012; Zhao et al., 2005; Mukherjee et al., 2012) and, when produced in high doses, they may arrest cell growth and induce apoptosis (Shao et al., 2004; Tabuchi et al., 2006). In *A. mellifera*, the HDACis activity has been linked to the regulation of immune and detoxification genes under stress from pesticides and *Nosema* (Hu et al., 2017), and, interestingly, to the epigenetic mechanism of royal jelly (Spannhoff et al., 2011). Importantly, similar to methylation patterns, emerging evidence suggests that information stored in nucleosomal hPTMs can be transmitted across cell divisions (Glastad et al., 2019).

#### 8.2.3 Non-coding RNAs (ncRNAs)

Non-coding RNAs (ncRNAs) are a varied class of RNAs that are not translated into proteins. Some of ncRNA products may have no specific function, but others play a key role in regulating cellular processes (Glastad et al., 2019). There are four types of ncRNAs that have been suggested to have a potential epigenetic effect: PIWI-interacting RNAs (piRNAs), microRNAs (miRNAs), small interfering RNAs (siRNAs), and long noncoding RNAs (lncRNAs). The small regulatory RNAs (piRNAs, miRNAs and siRNAs) form the RNAi (RNA interference) pathway, which is responsible for RNA-based antiviral immunity (see “immunity” section). RNAi is a post-transcriptional sequence-specific gene silencing mechanism that is involved in regulating gene expression in most organisms [reviewed by Ding (2010)]. Among them, miRNAs and siRNAs are major post-transcriptional gene expression regulators (reviewed by Richard et al. (2021)]. On the other hand, there is evidence that piRNAs and lncRNAs are linked to epigenetic effects that are particularly strong in insects [reviewed by Chambeyron and Seitz (2014)].

The small regulatory piRNAs are highly variable, short (21–35 nucleotides), single-stranded ncRNAs that are the primary small RNAs mediating chromatin modifications within insect genomes [reviewed by Chambeyron and Seitz (2014)]. In various organisms they are associated with PIWI proteins, which are part of Argonaute proteins [reviewed by Luteijn and Ketting (2013)]. They act to silence transposable elements (TEs), which are ubiquitous in insect genomes and can cause damage through aberrant recombination events and deleterious mutations. Interestingly, some piRNAs are maternally transmitted to offspring (Chambeyron and Seitz, 2014; Glastad et al., 2019). Another class of small regulatory RNAs are miRNAs, which are one of the best-studied classes of ncRNAs, although molecular evidence that they influence chromatin or are passed on through cell divisions in a truly epigenetic manner is still largely lacking in insects (Glastad et al., 2019). Finally, the siRNAs interact with proteins from the same families of miRNAs. It is important to note that, in addition to their epigenetic properties, siRNAs constitute an important antiviral defence mechanism in plants, fungi, nematodes, and arthropods, and the role of RNAi in honey bee antiviral defence has been demonstrated in laboratory-based experiments (McMenamin et al., 2018). Lastly, lncRNAs are transcripts of more than 200 nucleotides that are present in almost all eukaryotic organisms (Mercer et al., 2009) and share the potential for regulating gene expression at both transcriptional and post-transcriptional levels (Engreitz et al., 2016). They have been found to function in developmental processes in honey bees (Choudhary et al., 2021).

## 9 Immunity

**FIGURE 7:**
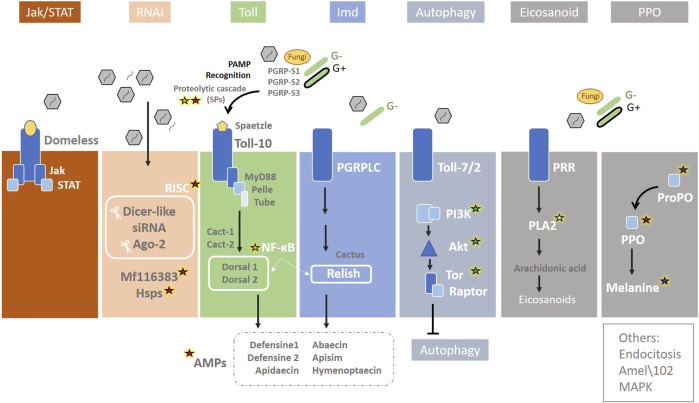
Immunity pathways in the honey bee. The main immunity pathways are represented together with the biotic stressors that are able to activate them: Jak/STAT (Janus Kinase/Signal Transducer and Activator of Transcription), RNAi (RNA interference), Toll *via* NF-ĸΒ (Nuclear Factor ĸΒ/Dorsal), Imd (immune deficiency) *via* NF-ĸΒ/Relish, JNK (c-Jun N-terminal kinase), MAPK (Mitogen-Activated Protein Kinases), autophagy, eicosanoid biosynthesis, endocytosis, and melanization and Prophenoloxidase (PPO). Genes involved in abiotic stress induced by climate change are highlighted: red, green, white, purple, and yellow stars indicate an association with heat, nutrition, humidity, UV, and oxidative stress, respectively. Redrawn based on (McMenamin et al., 2018).

Immunity is one of the main physiological mechanisms that regulate the overall survival of an organism, and, therefore, immune stress requires a substantial nutritional and energetic cost [reviewed by Lochmiller and Deerenberg (2000)]. The immune system of honey bees consists of a complex set of individual immune mechanisms and a special type of behavioural adaptations that protect against biotic and abiotic stress factors. As social insects, at the colony level, honey bees exhibit a collective defence mechanism known as “social immunity,” while at the individual level, they rely on cellular and humoral immune reactions. Within humoral immunity, antimicrobial peptides (AMPs) play a key role (Danihlík et al., 2015). At the molecular level, several immune pathways have been described in *A. mellifera*. These are mainly Jak/STAT (Janus Kinase/Signal Transducer and Activator of Transcription), RNAi (RNA interference), Toll *via* NF-ĸΒ (Nuclear Factor ĸΒ/Dorsal), Imd (immune deficiency) *via* NF-ĸΒ/Relish, JNK (c-Jun N-terminal kinase), MAPK (Mitogen-Activated Protein Kinases), autophagy, eicosanoid biosynthesis, endocytosis, melanization, and Prophenoloxidase (PPO) (McMenamin et al., 2018) (Figure 7).

Most of the stressors described in this review, directly or indirectly can modify the expression of genes related to at least one of these pathways (Table 1). For example, the first step when pathogens enter honey bee is the host recognition pathogen-associated molecular patterns from the invading microorganisms (PAMP recognition), which rapidly promote the activation of an SP cascade (Figure 7) [reviewed by McMenamin et al. (2018)]. Serine proteases, which have been described related to heat stress (see section 4), are also directly involved in immunity, as they are key in melanisation, wound healing, and phagocytosis stimulation by participating in the prophenoloxidase (PPO) activation pathway, RNAi, and SP proteolytic cascade in the Toll signalling [reviewed by Lu et al. (2014), McMenamin et al. (2018)] (Figure 3). Three genes of SP putative substrates, PPO, spätzle-1, and spätzle-2, are described in the *A. mellifera* genome (Zou et al., 2006). However, Phenoloxidase has been also linked to heat stress, existing different levels of enzymatic activity in workers, queens and drones exposed to the same stressful situation during development (Medina et al., 2020).

Regarding heat, there is a close relationship between the iRNAs and HSPs in honey bees. The RNAi pathway is initiated by Dicer-2 cleavage of viral dsRNA into 21–22 bp siRNAs, which are then loaded into Argonaute-2 (Ago2), the catalytic component of the RNA Induced Silencing Complex (RISC). Within this route, a putative serine/threonine cyclin-dependent kinase (MF116383) acts in non-specific dsRNA-mediated antiviral responses [reviewed by McMenamin et al. (2018)] (Figure 7). McMenamin et al. (2020) conducted an in-depth study of *A. mellifera,* comparing the transcriptomic response to heat shock and viral infections. The *mf116383* gene was the only immune-related gene of this pathway consistently induced by heat treatment alone and it has been suggested to serve as a point of cross-talk between the generalized antiviral immune response and the HSR in honey bees. On the other hand, the expression of *Hsc70-3*, *Hsc70-4*, and *Hsp90* was found to be positively correlated with *Dicer-like* (*Dcr-like)* and *Argonaute2 (Ago2)*, suggesting co-regulation of these genes (McMenamin et al., 2020). Cognate forms of *Hsp70* are involved in regulating the life cycle of various viruses, such as mediating attachment and endocytosis (Iša et al., 2004), penetration and uncoating, transcription and replication (Du et al., 2011), assembly and budding (Prange et al., 1999), and modulating autophagy (Beere, 2004). HSC70-4 is an important chaperone for the assembly of the RISC in *Drosophila* S2 cells and other flies (Dorner et al., 2006; Iwasaki et al., 2010). While further studies are needed to determine the mechanisms leading to the co-regulation of immune genes and HSP-encoding genes, it may be beneficial to co-regulate HSPs and HSP client proteins (Iwasaki et al., 2010). Meanwhile, heat stress in honey bee colonies has been linked to a reduction in virus and parasite infections [reviewed by Zhao et al. (2021)]. Finally, RNAi is not only key to antiviral defence and closely related to HSR, but may also act as epigenetic factors. The RNAi pathways is constituted by piRNAs, miRNAs and siRNAs, and is involved in regulating gene expression in most organisms [reviewed by Ding (2010)] (see “epigenetics” subsection).

Within the Toll pathway (Figure 7), NF-ĸB transcriptional factors are crucial in immunity, inflammatory response, cellular adhesion, differentiation, proliferation, autophagy, senescence, and apoptosis (Bonizzi and Karin, 2004). In *A. mellifera*, NF-ĸB factors Dorsal are encoded by two dorsal homologues genes, *Dorsal-1* and *Dorsal-2* (Evans et al., 2006). *Dorsal-1* produces two isoforms through alternative splicing (DORSAL-1A and DORSAL-1B) (Fan, 2001; Evans et al., 2006), and arguably this allows for a refinement of Toll-induced immune function. At this point, there is an important relationship between immunity and oxidative stress, consisting of both ROS influencing the activation of NF-κB pathway, and NF-κB pathway influencing the ROS levels by increasing the expression of antioxidant genes (Zhang et al., 2016). On the other hand, in adult bees, *Dorsal* genes are directly involved in the regulation of AMP genes together with Imd pathway (McMenamin et al., 2018), as *Defensine1* (Lourenço et al., 2018) and *Apidaecin* (Prisco et al., 2016). The activation of both Toll and Imd pathways also governs the expression of *Abaecin* (McMenamin et al., 2018). *Defensin* genes have been described as predictive markers of honey bee colony health linked to viral infections (Barroso-Arévalo et al., 2019). Two *Defensin* genes are described in honey bees: *Defensin1* and *Defensin2*. Sequences of these two genes revealed their different structure in a phylogenetic analysis, with *Defensin1* forming a clade with two other hymenopteran defensins, whereas *Defensin2* grouped with coleopteran defensins (Klaudiny et al., 2005). In contrast to other invertebrates, heat shock in *A. mellifera* represses the expression of certain antimicrobial peptide genes such as *Hymenoptaecin*, *Defensin1* and *Abaecin* (McKinstry et al., 2017). This may be due to an energetic trade-off in the honeybee between cellular mechanisms for maintaining proteostasis and the immune response, as both are energetically costly (Powers and Balch, 2013).

The link between nutrition and immunity is well known. In fact, “nutritional immunology” is a discipline that studies the composition of the diet to optimise the immune response (Ponton et al., 2011). The interaction between nutritional and immune pathways is as wide as complex. For example, miRNAs have been described as acting on the insulin and TOR pathways (Shi et al., 2015), while PiP3, AKT and TOR inhibit autophagy (Figure 4) (McMenamin et al., 2018). On the other hand, inadequate nutrition stimulates adenylate kinase (Ak6) (involved in stress-induced pathways), NF-ĸB, and genotoxic/non-genotoxic stress (Hou et al., 2008; Barrio et al., 2014; Hasygar and Hietakangas, 2014; Corona et al., 2023). Furthermore, VG is involved in immunity in honey bees (Amdam et al., 2004). Recently, Azzouz-Olden et al. (2018) conducted an in-depth study comparing well- and poorly-fed honey bees between two groups comprising infected and non-infected individuals with the microsporidium *Nosema apis*. The authors showed that poorly fed and infected individuals underwent a significant upregulation of some genes related to the Toll pathway, such as *Sph*, *serpine-1* (*Nec*), *Tub*, *Cactus*, *PGRP-S3* and AMP genes (like *Apisimin*). On the other hand, during melanisation (see section 6), melanins are produced by the enzymatic oxidation of tyrosine by tyrosinase, which is the enzyme’s main activating system (PPO). Tyrosinase depends upon tyrosine, which derives from phenylalanine, an essential amino acid that can only be obtained from ingested food. In addition, the defensive compounds produced by the bees are rich in nitrogen, requiring a significant investment of their resources. Hence, the PO response involves a high energetic cost for each bee (González-Santoyo and Córdoba-Aguilar, 2012; Negri et al., 2019).

It is important to note that the PLA2 protein, involved in stress induced by humidity, is involved in immunity through the arachidonic acid release in the eicosanoid pathway (McMenamin et al., 2018).

## 10 Conclusion

Predicting how climate change will impact honey bees is complex and challenging. It will not only directly affect individual bees and colonies but also compound existing stressors, alter the ecosystems they inhabit, and introduce new and additional pressures. Higher temperatures, changes in humidity, and increased exposure to UV radiation will not only affect bee biology but also contribute to colony stress, alter plant flowering (and thus bee food resources), and influence disease dynamics. These factors can both benefit the pathogens under the new conditions and weaken the host organisms. In this review, we aimed to summarise a guide of molecular targets potentially linked to climate change stress, along with the cellular pathways directly involved or related. Genetic studies are a valuable tool for addressing these issues, and understanding the related mechanisms and pathways is essential for correctly interpreting molecular insights. Knowing which genes are involved in a specific pathway, what they are like, what their transcripts are like, how they express under stress, and how they affect other pathways, provides a basis for future studies aimed at measuring not only the degree of stress in honey bees but also its consequences for their cell biology.

## Glossary

AChE: Acetylcholinesterase
Ace: Acetylcholinesterase gene
AMPs: antimicrobial peptides
AO: Antioxidant
AS: Alternative splicing
CpG: Cytosine-Guanine dinucleotide
CRH-BP: Cortico-releasing hormone-binding protein
HDAC: Histone Deacetylase
HDACis: Histone Deacetylase inhibitors
DNMTs: DNA methyltransferases
hPTMs: Histone post-translational modifications
HS: Heat Shock
HSC: Heat Shock Protein cognate form
HSF: Heat Shock Factor
HSE: Heat Shock Element
HSR: Heat Shock Response
HSP: Heat Shock Proteins
FOXO: Forkhead box O subfamily of genes
GSH: glutathione
GST: glutathione S-transferase
IIS: Insulin/insulin-like growth factor signalling
RNAi: RNA of interference
JH: juvenile hormone
ncRNAs: Non-coding RNAs
NF-Y: Nuclear Factor Y
OA: Octopamine
PPO: prophenoloxidase
PO: phenoloxidase
RH: Relative humidity
ROS: reactive oxygen species
sHSP: small Heat Shock Proteins
SP: Serine Protease
STK: Serine/threonine kinase
SNP: Single Nucleotide Polymorphism
SOD: Superoxide dismutase
TA: Tyramine
TEs: Transposable elements
TOR: Target of Rapamycin
TRX: thioredoxin
UV: Ultraviolet
VG: Vitellogenin
ZFP: Zinc Finger Protein
