# Supplementary material for: Climate change-induced stress in the honey bee Apis mellifera L.- a genetic review
Source: Front Physiol. 2025 Sep 17;16:1623705. doi: 10.3389/fphys.2025.1623705 (PMC12484183; doi:10.3389/fphys.2025.1623705)
Supplement: Supplementary file 1 [file Supplementaryfile1.pdf]

Supplementary Tables.

Each of the genes has been assigned to the group described in the review. Gene location is based on the *Amel_HAv3.1* annotation release (GCF_003254395.2). Descriptions and gene names are according to NCBI (accessed January 2025).

S.I. Table 1. Candidate search terms focusing on insects.

Section / Subsection	Research question	Candidate search terms
**1**	General	"Climate change" and "honey bees" OR "*Apis mellifera*" OR "insects", OR "pollinators", "adaptation", "*Apis mellifera* genome", "phenotypic plasticity", "epigenetics", "splicing"
**2**	Methods	PubMed, Scopus, Google Scholar, Web of Science
**3**	Stress	"Stress" in "insects" OR "bees" OR "honey bees" OR "*Apis mellifera*"
**4**	Temperature	"Climate change" and "Honey bees" OR "bees" OR "*Apis mellifera*", OR "*A. mellifera* subspecies", "pollinators", "temperature", "heat stress", "heat tolerance"
4.1	HSR	"Heat hock response", "bees" OR "honey bees" OR "*Apis mellifera*", "Heat shock factor", "insects", "*Drosophila melanogaster*", "alternative splicing" OR "AS"
4.1.1	HSP	"Heat Shock Proteins" in "*Apis mellifera*" OR "Honey bees", "HSP110", "HSP90", "HSP70", "HSP60" OR "chaperonins", "HSP40" OR "DnaJ", "HSP10" OR "small HSPs" OR "protein lethal (2) essential for life", "cochaperones", "alternative splicing" OR "AS"
4.2.1	NFY	"Nuclear Factor Y" OR "NF-Y" OR "Heme Activator Protein" OR "HAP" OR "CCAAT-Binding Factor" OR "CBF" OR NF-YA, OR NF-YB, OR NF-YC, "heat stress" in "bees" OR "honey bees" OR "*Apis mellifera*", "insects", "*Drosophila melanogaster*", "alternative splicing" OR "AS"
4.2.2	ZFP	"Zinc finger proteins" OR "ZFP", "KRAB" OR "Kruppel-associated box", in "bees" OR "honey bees" OR "*Apis mellifera*" OR "insects", "heat stress", "alternative splicing" OR "AS"
4.2.3	SP	"Proteases", "Serine proteases" OR "SP", "serpin" OR "Serine Protease Inhibitors" in "bees" OR "honey bees" OR "*Apis mellifera*" OR "*Apis cerana*" OR "insects", "heat stress", "immunity", "alternative splicing" OR "AS"
4.2.4	Antoxidant enzymes	"Oxidative stress", "Antioxidant enzymes", "redox", "Antioxidant genes" in "*Apis mellifera*" OR "bees" OR "honey bees", "*Drosophila melanogaster*", "heat stress", "DnaJA1" OR "HSP40", "alternative splicing" OR "AS"
4.2.5	AChE	"Acetylcholinesterase" OR "AChE" in "insects" OR "*Apis mellifera*", OR "bees" OR "honey bees", "Ace1, "Ace2", Heat stress", "HSR", "pesticides", "alternative splicing" OR "AS"
4.2.6	CRH-BP	"Corticotropin-releasing hormone-binding protein" OR "CRH-BP" in "heats stress" in "bees" OR "honey bees" OR "*Apis mellifera*" OR "*Apis cerana*" OR "insects", "alternative splicing" OR "AS"
**5**	Humidity	"Climate change", "precipitation patters", "Relative humidity" in "bees", OR "honey bees" OR "*Apis mellifera*" OR "*Apis mellifera* subspecies", "heat stress", "phospholipase A2 " OR "Pla2", "antifreeze protein " OR "Afp", "alternative splicing" OR "AS"
**6**	Ultraviolet exposure	"Climate change", "Ultraviolet" OR "UV" exposure, "UV radiation" and "DNA damage" in "bees" OR "honey bees" OR "*Apis mellifera*", "insects", "*Drosophila melanogaster*", "melanin", "heat stress", "oxidative stress", "immunity", "PPO" OR "Prophenoloxidase", "small HSPs", "CRH-BP", "alternative splicing" OR "AS"
**7**	Nutrition	"Climate change" in "honey bees" OR "*Apis mellifera*" OR "insects", OR "pollinators", "nutrition", "nutritional stress", "thermal stress", "phenology", "floral resources", "survival, "adaptation", "insulin/insulin-like growth signalling" OR "IIS", "Target of Rapamycin" OR "TOR", "Forkhead box O subfamily" OR "FOXO", "alternative splicing" OR "AS"

7.1	Phenolamines	“Nutritional stress”, “starvation genes”, “tyramine”, “octopamine”, “adenylyl cyclase”, “alternative splicing” OR “AS”
7.2	Storage proteins	“Vitellogenin” OR “Vg”, “nutrition” in “honey bees” OR “*Apis mellifera*”, “heat stress”, “oxidative stress”, “immunity”, “Vg genes”, “hexamerins”, “hex70a”, “hex70b”, “hex70c”, “hex110”, “alternative splicing” OR “AS”
8.1	Alternative splicing	“Splicing”, “alternative splicing” OR “AS”, “RNA processing”, “splicesome”, “biotic and abiotic stress”
8.2	Epigenetics	“Epigenetics”, “phenotypic plasticity”, honey bees” OR “*Apis mellifera*”, “*Apis mellifera* genome”, “thermal stress”, “adaptation”
8.2.1	DNA methylation	“Epigenetics”, “methylation”, “epitranscriptome” and “stress” in “bees” OR “honey bees” OR “*Apis mellifera”, “*insects*”*, “*Drosophila melanogaster”*, “DNA methyltransferases” OR “DNMTs”, “DNMT1”, “DNMT3”, “alternative splicing” OR “AS”
8.2.2	Histone post-translational modifications	“Epigenetics”, “Histone post-translational modifications” OR “hPTMs”, “nucleosome”, “deacetylases” OR “HDAC”, “HDAC inhibitors” OR “HDACis”, “stress” in “bees” OR “honey bees” OR “*Apis mellifera”*
8.2.3	ncRNAs	“Epigenetics”, “Non-coding RNAs” OR “ncRNAs”, “PIWI-interacting RNAs” OR “piRNAs”, “microRNAs” OR “miRNAs, “small interfering RNAs” OR “siRNAs”, “long noncoding RNAs” OR “lncRNAs”, in “bees” OR “honey bees” OR “*Apis mellifera”, “insects”*
**9**	Immunity	“Immunity” in “bees” OR “honey bees” OR “*Apis mellifera”*, “*Drosophila melanogaster”,* “stress”, “HSR”, “Toll signalling”, “SP cascade”, “NF-κB factors”, “DORSAL-1A”, “DORSAL-1B”, “antimicrobial peptides” OR “AMPs”, “HSPs”, “iRNA”, “RNA Induced Silencing Complex” OR “RISC”, “MF116383”, “Defensine1”, “Defensin2”, “Apidaecin”, “Abaecin”, “Hymenoptaecin”, “nutrition”, “alternative splicing” OR “AS”

S.I. Table 2. Genes related to Heat Shock Response (HSR).

Group	Description	Gene name	Aliases	Location
HSF	Heat shock factor protein; heat shock factor protein 1	LOC411854	Hsf, GB52628	LG10: 8178315-8188320, complement
HSF	Heat shock factor binding protein 1-like	LOC725609	GB16802, GB45505	LG1: 19786056-19786958, complement
HSF	Heat shock factor 2-binding protein	LOC100577896	GB46682	LG5: 5399561-5403165
HSF	Heat shock transcription factor, Y-linked-like	LOC102656395		LG1: 4746833-4749201
HSP90	Heat shock protein 90	Hsp90	GB14758, GB40976, Hsp83	LG7: 9550759-9553976
HSP90	Heat shock protein 83	LOC411700	GB45495	LG1: 20112796-20115650, complement
HSP70	Heat shock protein Hsp70Ab-like	LOC410620	GB19503, GB50609	LG15: 5231544-5235275
HSP70	Heat shock protein 70Cb, 97 kDa heat shock protein; heat shock 70 kDa protein 4; heat shock protein 70Cb ortholog	Hsc70Cb	GB50730	Chromosome LG2, NC_037639.1 (6762713..6771073, complement)
HSP70	TNF receptor associated protein 1; heat shock protein 75 kDa, mitochondrial	Trap1	GB46339, LOC550968	LG6: 11859277-11862923, complement
HSC70	Heat shock protein 70 cognate 3; heat shock protein cognate 3; heat shock 70 kDa protein cognate 3	Hsc70-3	GB15016, GB49117	LG3: 3212644-3216513, complement
HSC70	Heat shock protein cognate 4	Hsc70-4	GB14852, GB40866	LG1: 5356800-361132, complement
HSC70	Heat shock protein 70 cognate 5	Hsc70-5	GB19860, GB42297	LG1: 26843272-26847829
HSP60	Heat shock protein 60A	LOC409384	GB54372	LG16: 1230961-1234962
HSP10	10 kDa heat shock protein, mitochondrial	LOC552531	GB54343	LG16: 1228522-1229993, complement
HSP40	dnaJ homolog subfamily C member 16 l(3)80Fg; dnaJ homolog subfamily C member 16	l(3)80Fg	GB47667, LOC412118	LG1: 2083650-2089200, complement
HSP40	dnaJ homolog subfamily C member 8	LOC410294	GB49666	LG11: 1330165-1332218, complement
HSP40	dnaJ homolog subfamily C member 28	LOC551875	GB41234	LG3: 13083131-13085110, complement
HSP40	dnaJ homolog subfamily B member 13-like	LOC727642	GB51034	LG14: 5418324-5420911, complement
HSP40	Tetratricopeptide repeat protein 2; dnaJ homolog subfamily C member 7	Tpr2	GB41796, LOC410037	LG8: 7792065-7795027, complement
HSP40	dnaJ homolog subfamily B member 6	LOC408966	GB41858	LG8: 7608846-7621581
HSP40	receptor mediated endocytosis 8; dnaJ homolog subfamily C member 13	Rme-8	LOC411060, GB52306,	LG5: 1919443-1929829
HSP40	dnaJ homolog subfamily C member 5; cysteine string protein	LOC724965	Csp, GB49934	LG15: 9452670-9468801, complement
HSP40	dnaJ homolog subfamily C member 21	LOC100578360	GB54992	LG1: 24634192-24636640, complement
HSP40	DnaJ heat shock protein family (Hsp40) member A4-like; dnaJ homolog subfamily A member 1, DnaJ-like-2	Droj2	LOC724368, GB46569	LG4: 9927392-9932492, complement
HSP40	dnaJ homolog subfamily C member 17	LOC552846	GB45636	LG5: 13823766-13825772, complement
HSP40	dnaJ homolog shv, dnaJ homolog subfamily B member 11	Shv	GB51659, LOC552223	LG8: 12396314-12398408, complement
HSP40	dnaJ homolog subfamily C member 2	LOC413209	GB55532	LG2: 15662820-15667773, complement
HSP40	dnaJ homolog subfamily C member 11	LOC409991	GB43856	LG8: 6967788-6970453
HSP40	TM2 and DnaJ domain-containing protein wurst; dnaJ homolog subfamily C member 22	wus	GB45851, LOC410730	LG1: 10469530-10472687, complement
HSP40	dnaJ homolog subfamily C member 1	LOC552151	GB43868	LG8: 7166574-7168776, complement
HSP40	dnaJ homolog subfamily C member P58IPK; dnaJ homolog subfamily C member 3	P58IPK	GB48812, LOC413441	LG10: 11289770-11293337, complement

HSP40	dnaJ homolog subfamily B member 12	LOC413120	GB47651	LG16: 4639240-4643300, complement
sHSP	protein lethal (2) essential for life	LOC724488	GB45913	LG2: 4837466-4838343
sHSP	protein lethal (2) essential for life	LOC410857	GB49775	LG2: 11826715-11837150
sHSP	protein lethal (2) essential for life	LOC412197	GB47475	LG1: 15118751-15120554
sHSP	protein lethal (2) essential for life	LOC724405	GB11383	LG2: 4831304-4832577
sHSP	protein lethal (2) essential for life	LOC724367	GB45910	LG2: 4828926-4829893
sHSP	protein lethal (2) essential for life	LOC724274	GB10397	LG2: 4823146-4824181
sHSP	protein lethal (2) essential for life	LOC724449	GB45912	LG2: 4833936-4835076
sHSP	Heat shock protein beta-1; alpha-crystallin B chain	LOC408875	GB53550	LG6: 14242111-14268326

S.I. Table 3. Other genes involved in heat stress.

Group	Description	Gene name	Aliases	Location
NF-Y	Nuclear factor Y-box A; nuclear transcription factor Y subunit alpha	Nf-YA	LOC725757, GB50732	LG2: 6754032-6756801, complement
NF-Y	Nuclear factor Y-box B; nuclear transcription factor Y subunit B-1	Nf-YB	LOC411193, GB48127	LG7: 13760993-13762541
NF-Y	Nuclear factor Y-box C; nuclear transcription factor Y subunit gamma	Nf-YC	LOC408615, GB54831	LG1: 5056876-5060346, complement
NF-Y	Nuclear transcription factor Y subunit gamma-like	LOC113218635		LG3: 9362453-9366633, complement
ZFP	Zinc finger protein 271; zinc finger protein 665, zinc finger protein 880	LOC409470	GB54040	LG11: 7129964-7147480, complement
ZFP	Zinc finger protein 271	LOC727269	GB55976	LG10: 409400-412566, complement)
ZFP	Zinc finger protein 271	LOC100578924		LG15: 7619194-7621734, complement
ZFP	Zinc finger protein 271	LOC725279	GB45817	LG1: 10908020-10909835, complement
ZFP	Zinc finger protein 37 homolog	LOC102654585		LG9: 10673548-10675272, complement
ZFP	Zinc finger protein 37	LOC102654889		LG4: 6730347-6733926
ZFP	Zinc finger protein 239	LOC102654736		LG4: 6706110-6709396
ZFP	Zinc finger protein 239-like	LOC102654597		LG16: 7175555-7177626
ZFP	Zinc finger protein 776-like	LOC102654541		LG9: 10672197-10673067, complement
ZFP	Kruppel homolog 1; Krueppel homologous protein 1; kruppel-like protein 1	Kr-h1	GB45427	LG6: 16501760-16510451, complement
ZFP	Zinc finger protein 62; zinc finger protein 37	LOC413515	GB54650	LG15: 2822067-2825878, complement
ZFP	Zinc finger protein 628-like; protein bric-a-brac 1-like; maternal gene required for meiosis	mamo	LOC102654975, GB11420, GB50425	LG4: 8378760-8475988
ZFP	Zinc finger protein 431; zinc finger protein 135	LOC100578743	GB52746	LG14: 9098326-9101919, complement
SP	Uncharacterized LOC726286; Serine protease 1	LOC726286	GB45701, SP1, cSP1	LG5: 13459653-13465020
SP substrate	Uncharacterized LOC100577844; Spaetzle	LOC100577844	Spz, GB52631	LG10: 8159836-8163671, complement
SP substrate	Neurothropin-1; spatzle 2	LOC725353	GB40604, spz2	LG12: 7115679-7126064, complement
SP substrate	Spaetzle domain-containing protein 3; protein spaetzle 3	Spz3	LOC726201, GB51155	LG10: 10640626-10652345
SP substrate	Spaetzle domain-containing protein 4; protein spaetzle 4	Spz4	LOC100578719	LG10: 11677194-11684023
SP substrate	Spaetzle 5; protein spaetzle 5	Spz5	LOC725541, GB52553	LG12: 4235637-4253028, complement
SP substrate	Spaetzle domain containing protein 6; uncharacterized protein LOC408317; spatzle 6	Spz6	LOC408317, GB45218	LG11: 14399987-14404363
AchE	Acetylcholinesterase 1; acetylcholinesterase	LOC410270	ACE1, AChE-1, GB43191	LG11: 15756848-15791185
AchE	Acetylcholinesterase 2; NP_001035320.1	AChE-2	GB14873, GB41856, ACE2, Ache	LG8: 7426943-7561836
Crh-BP	Corticotropin releasing hormone binding protein	Crh-BP	GB14094, GB40439	LG8: 2187253-2195465, complement

Cat	Catalase; NP_001171540.1; XP_026296889.1	Cat	GB11648, GB41427	LG6: 8659532-8670508
SOD	Superoxid dismutase 1; CuZn superoxide dismutase; NP_001171498.1	Sod1	GB10133, GB47880	LG8: 529896-531140
SOD	Superoxid dismutase 2; Mn Sod; Mn superoxide dismutase; superoxide dismutase 2, mitochondrial; NP_001171519.1	Sod2	GB14346, GB45099	LG11: 11069598-11071864
SOD	Related to Sod; uncharacterized protein LOC413369; copper transporter superoxide dismutase	Rsod	CT-SOD, GB40203, LOC413369	LG12: 889125-894963
SOD	Superoxid dismutase [Cu-Zn], chloroplastic-like; superoxide dismutase 3	LOC113218 958	Sod[Cu-Zn]-like	LG8: 1563472-1565310
POD	Peroxidase	LOC408953	GB49688	LG7: 885875-917307, complement
POD	Peroxidase, NP_001207416.1	LOC724541	GB19100, GB42296	LG1: 26835755-26842314
POD	Peroxidase	LOC551544	GB49689, Pxd	LG7: 848684-858060, complement
GTPX	Glutathione peroxidase-like 1; putative thioredoxin peroxidase	Gtpx1	GB14138, GB47478, Gtpx-1	LG1: 15149964-15153267
POD	Peroxidase homolog; chorion peroxidase	Cysu; LOC412013	Eh, GB49646	LG4: 449832-502649, complement
GTPX	Probable phospholipid hydroperoxide glutathione peroxidase; glutathione peroxidase-like 2	LOC726269	GB48634, Gtpx2	LG5: 8255952-8258228, complement
POD	Myeloperoxidase	LOC413054	GB43935	LG11: 738073-743171
TPX	Thioredoxin peroxidase 4; peroxiredoxin-like protein	Tpx-4	GB10498, GB41413	LG9: 2864435-2869887, complement
TPX	Thioredoxin peroxidase 3	Tpx-3	GB10972, GB50555	LG15: 4365484-4366905
TPX	Peroxiredoxin 2; peroxiredoxin 1; thioredoxin peroxidase 1	Prx2	GB40232, Tpx-1, LOC409954	LG12: 988272-991191, complement
TPX	Peroxiredoxin-6, thioredoxin peroxidase 5	LOC411852	GB52120, Tpx-5, Tpx-6	LG12: 8689647-8691836, complement
TPX	Peroxiredoxin-5, mitochondrial	LOC552429	GB43999, Prx5	LG4: 10159586-10161094
POD	Peroxidasin	LOC413025	GB44808, Pxn	LG1: 11075510-11089568, complement
TRX	Thioredoxin reductase 1; NP_001171496.1; NP_001171497.1; XP_006563264.1	Trxr1	GB14972, GB40718, Trxr-1	LG1: 5454493-5460515
TRX	Uncharacterized LOC725664, thioredoxin 1-like 2	LOC725664	GB55856, Trx1-like2	LG3: 5035114-5043770, complement
TRX	Thioredoxin, mitochondrial; thioredoxin 1	LOC410120	GB52684, Trx-1	LG14: 8115629-8116817
TRX	Thioredoxin domain-containing protein 17-like	LOC102656 890		LG3: 4528906-4531784, complement
TRX	Thioredoxin domain-containing protein 9	LOC551696	GB41973	LG8: 11698104-11699262
TRX	Thioredoxin reductase-like selenoprotein T homolog CG3887; selT-like protein	LOC550992	GB54973	LG1: 25087375-25088764, complement
TRX	Thioredoxin domain-containing protein	LOC411255	GB54503	LG8: 5275233-5277352, complement
TRX	Thioredoxin domain-containing protein 12-like	LOC113219 403		LG1: 11764475-11765275
TRX	Endoplasmic reticulum protein 44	ERp44	GB48215, Trx1-like3, LOC552191	LG13: 3229417-3233396
TRX	Thioredoxin-like protein Dim1; thioredoxin-like protein 4A	LOC551974, Dim1	GB40801	LG1: 6571641-6572750, complement
TRX	Thioredoxin-like protein 1; thioredoxin 1-like 1	LOC550734	GB43742, Trx1-like1	LG14: 6356826-6359193
TRX	Thioredoxin, mitochondrial; thioredoxin, mitochondrial pseudogene	LOC100578 002		LG11: 10575866-10576484, complement
TRX	Thioredoxin domain-containing protein pretaporter, thioredoxin	prtp	GB51282, LOC408557	LG16: 3180378-3182833

	domain-containing protein 5 homolog			
TRX	Thioredoxin-related transmembrane protein 2 homolog	LOC411391	GB11970, GB43843	LG8: 6610309-6611952
TRX	Thioredoxin-2	LOC409451	GB48574, Trx-2	LG6: 6611748-6613808
TRX	Thioredoxin-related transmembrane protein 1	LOC414042	GB48928	LG1: 20568402-20569981, complement
TRX	Thioredoxin domain-containing protein 11	LOC410121	GB52686	LG14: 8120936-8125701
TRX	Apoptosis-inducing factor 3; thioredoxin reductase 3	LOC413958	GB49242, Trxr-3	LG7: 3509339-3512955
TRX	Thioredoxin domain-containing protein 17; clot	LOC412420	GB46904, cl	Not in annotation release Amel_HAv3.1
GST	Glutathione S-transferase S4; glutathione S-transferase HP19	GstS4	GB14372, GB49545, hp19	LG4: 5016633-5017794, complement
GST	GST-containing FLYWCH zinc-finger protein; glutathione S-transferase 1-1; glutathione S-transferase 1; glutathione S-transferase U1	gfzf	GB49614, GstU1, LOC725942	LG4: 5649865-5652928
GST	Glutathione S-transferase D1	GstD1	GB18045, GB50265, Gst1	LG15: 9011536-9016677
GST	Glutathione S-transferase theta-3; glutathione S-transferase T1; glutathione S-transferase theta-1	LOC552314	GB42961, GstT1	LG6: 12181659-12184617, complement
GST	Pyrimidodiazepine synthase; glutathione S-transferase O2; glutathione S-transferase omega-1	LOC726823	GB51243, GstO2	LG7: 4729272-4731424, complement
GST	Glutathione S-transferase S1	GstS1	GB16959, GB48905	LG4: 1918073-1921443
GST	Glutathione S-transferase, C-terminal domain containing protein	Gstcd	GB19050	LG2: 14738981-14741891, complement
GST	Microsomal glutathione S-transferase 1	LOC410837	GB55590, GST-mic1, Mgstl	LG2: 14723583-14724378, complement
GST	Probable maleylacetoacetate isomerase 2; glutathione S-transferase Z1	LOC411088	GB48672, GstZ1	LG5: 8430697-8433098
GST	Pyrimidodiazepine synthase; glutathione S-transferase O1	LOC552118	GB44803, GstO1	LG1: 11125422-11130015, complement
GST	Glutathione S-transferase 1-1-like	LOC102656383		LG15: 9018465-9020364
SOD	Copper chaperone for superoxide dismutase	Ccs	GB46896, LOC552629	LG3: 4477094-4479450
MsrA	Methionine sulphoxide reductase A; methionine sulfoxide reductase A; NP_001171518.1; XP_006571522.1; XP_016766477.1; XP_026295502.1	MsrA	GB10196, GB55004	LG1: 24242210-24245935, complement
MsrB	Methionine sulfoxide reductase SelR; methionine-R-sulfoxide reductase B1; methionine sulfoxide reductase B	SelR	GB48797, MsrB, LOC724494	LG10: 11424810-11430693, complement
GRX	Glutaredoxin-C4; glutaredoxin 1	LOC727309	Grx1, GB41663	LG14: 10573041-10574239
GRX	Glutaredoxin-related protein 5, mitochondrial; glutaredoxin 2	LOC552835	Grx2, GB45288	LG11: 15244173-15245244
GRX	Uncharacterized LOC411159; glutaredoxin-like 1	LOC411159	GB52955; Grx-like1	LG6: 10268943-10311840, complement

S.I. Table 4. Genes related to stress by humidity.

Group	Description	Gene name	Aliases	Location
Pla2	Phospholipase A2; allergen Api m 1; allergen Api m I; phosphatidylcholine 2-acylhydrolase; NP_001011614.1	Pla2	GB13351, bvPLA2, GB48228	LG13: 3562941-3564376
Pla2	Acidic phospholipase A2 PA4; uncharacterized protein LOC409277; phospholipase A2-2.2	LOC409277	PLA2-2.2, GB46277	LG7: 1312285-1320042, complement
Pla2	Phospholipase A2-like	LOC724436	GB19761, GB44367	LG7: 12181630-12183527, complement
Pla2	Group XIIA secretory phospholipase A2	LOC409614	GB41664	LG14: 10574488-10577092
Pla2	Uncharacterized LOC409307; phospholipase A2-2.4	LOC409307	PLA2-2.4, GB44868	LG11: 15341730-15343741, complement
Pla2	Uncharacterized LOC552242; LOW QUALITY PROTEIN: uncharacterized protein LOC552242; phospholipase A2-1	LOC552242	PLA2-1, GB40344	LG8: 3784750-3787316, complement
Pla2	Calcium-independent phospholipase A2-gamma	LOC726656	GB52882	LG7: 401534-406286, complement
Pla2	Group XV phospholipase A2	LOC552091	GB55297	LG1: 14352996-14355214, complement
Pla2	Calcium-independent phospholipase A2 VIA; 85/88 kDa calcium-independent phospholipase A2	iPLA2-VIA	LOC410570, GB41694	LG14: 10427732-10431291, complement
Pla2	Phospholipase A2 activator protein; phospholipase A-2-activating protein	Plap	LOC409219, GB41825	LG8: 7274978-7278903, complement
Pla2	1-acylglycerol-3-phosphate O-acyltransferase Pnpla3; patatin-like phospholipase domain-containing protein 3	LOC551055	GB41447	LG6: 9083191-9094373
Afp	Antifreeze protein Maxi; maxi-like antifreeze protein	LOC102654146		LG2: 346229-353976, complement

S.I. Table 5. Genes related to stress by UV exposure.

Group	Description	Gene name	Aliases	Location
PPO	Phenoloxidase subunit A3; prophenoloxidase; NP_001011627.1	PPO	ppoq, GB18313, GB43738	LG14: 6001332-6004996
DnaJA	DnaJ heat shock protein family (Hsp40) member A4-like; dnaJ homolog subfamily A member 1; DnaJ-like-2	Droj2	GB46569, LOC724368	LG4: 9927392-9932492, complement
DnaJB	DnaJ homolog subfamily B member 12	LOC413120	GB47651	LG16: 4639240-4643300, complement
DnaJC	DnaJ homolog subfamily C member 8	LOC410294	GB49666	LG11: 1330165-1332218, complement
Crh-BP	Corticotropin releasing hormone binding protein	Crh-BP	GB14094; GB40439	LG8: 2187253-2195465, complement

S.I. Table 6. Genes related to nutritional stress.

Group	Description	Gene name	Aliases	Location
FOXO	Forkhead box protein sub-group O; forkhead box protein O	foxo	GB48301, LOC727091	LG7: 5302778-5523561
ILP	Insulin-like peptide 2; bombyxin	ILP-2	GB10174, GB43560	LG14: 5016214-5017942, complement
IGF	Insulin-like growth factor I; uncharacterized protein LOC100577028	LOC100577028		LG8: 4175629-4185151, complement
IGF	IGF-II mRNA-binding protein; insulin-like growth factor 2 mRNA-binding protein 1	Imp	LOC410398, GB52056	LG12: 10759854-10829510, complement
InR	Insulin-like peptide receptor; insulin receptor B; insulin-like receptor	LOC411297	GB53353, IR-B, InR, InsR-A	LG9: 11585175-11612673
InR	Insulin-like receptor-like; insulin receptor 2	InR-2	GB55425	LG2: 14542801-14593835
Chico	Insulin receptor substrate 1 chico; insulin receptor substrate 1-B	chico	IRS, InRS, GB49911, LOC408438	LG13: 10087421-10098733, complement
PI3K	Phosphatidylinositol-4-phosphate 3-kinase catalytic subunit Pi3K68D; phosphatidylinositol 4-phosphate 3-kinase C2 domain-containing subunit beta; phosphotidylinositol 3 kinase 68D ortholog	Pi3K68D	GB48994	LG1: 7924475-7934823
PI3K	Phosphatidylinositol 3-kinase 92E; phosphatidylinositol 4,5-bisphosphate 3-kinase catalytic subunit delta isoform; phosphatidylinositol 4,5-bisphosphate 3-kinase catalytic subunit	Pi3K92E	GB43074	LG11: 16317895-16326171, complement
PI3K	Phosphatidylinositol 3-kinase 59F; phosphatidylinositol 3-kinase catalytic subunit type 3; phosphotidylinositol 3 kinase 59F ortholog	Pi3K59F	GB48601	LG6: 7113686-7118094
PI3K	Phosphatidylinositol 3-kinase regulatory subunit alpha; phosphatidylinositol 3-kinase regulatory subunit alpha; uncharacterized protein LOC408577; phosphotidylinositol 3 kinase 21B ortholog	Pi3K21B	GB42200	LG1: 18893490-18924923
Tor	Serine/threonine-protein kinase Tor; serine/threonine-protein kinase mTOR; target of rapamycin	Tor	GB44905	LG11: 14862031-14870779, complement
Tor	Rapamycin-insensitive companion of Tor; rapamycin-insensitive companion of mTOR	Rictor	GB52079	LG12: 10017631-10023675, complement
AKT	AKT serine/threonine protein kinase; RAC serine/threonine-protein kinase	Akt	Akt1, GB43135	LG11: 15629173-15633129, complement
AKT	AKT-interacting protein	LOC552831	GB45656	LG5: 13600634-13602794, complement
AKT	Proline-rich Akt substrate 40 kDa; uncharacterized protein LOC551512	PRAS40	lobe; GB48086	LG6: 2959802-2963324, complement
Adenylyl cyclase	Adenylate cyclase type 8-like; adenylyl cyclase 78C; adenylyl cyclase; NP_001314893.1; XP_016770050.2; XP_026298874.1; XP_026298875.1; XP_026298876.1	Adcy8	ac8, GB54593, LOC726262	LG10: 4801592-4815632, complement
Adenylyl cyclase	Adenylyl cyclase-associated protein 1	capt	GB40592, LOC410158	LG10: 4648774-4666654, complement
Adenylyl cyclase	Adenylyl cyclase X D; adenylate cyclase type 2; adenylyl cyclase	ACXD	GB48102, ac2, LOC551461	LG7: 13377789-13390880
Adenylyl cyclase	Adenylate cyclase type 2 Ac76E; adenylate cyclase type 2; adenylyl cyclase 76E ortholog	Adcy2	GB42675, LOC552216	LG9: 7550543-7558077, complement
Adenylyl cyclase	Adenylate cyclase 3; NP_001071276.1; XP_006566310.1; XP_016770150.1	Ac3	Amac3, GB11637, GB45150	LG11: 12956997-12973602
Adenylyl cyclase	Adenylate cyclase type 6	LOC726514	GB41921	LG8: 8680214-8780078
OA	Octopamine receptor in mushroom bodies; octopamine receptor 1	Oamb	Oar, GB11266, GB52910, Oa1	LG15: 3310369-3326664, complement

OA	Octopamine receptor beta-2R; octopamine beta-type receptor 2	LOC412896	GB49696, Octbeta2, Octbeta2R	LG7: 564310-742348, complement
OA	Octopamine receptor beta-3R; octopamine beta-type receptor 3; octopamine beta-type receptor 4	LOC412994	GB52879, Octbeta3, Octbeta4	LG7: 413064-522092
OA	Octopamine receptor beta-1R; octopamine beta-type receptor 1; octopamine receptor 2	LOC413698	Oa2, GB43263, Octbeta1	LG7: 228383-289772
Vg	Vitellogenin	Vgn	GB13999, GB49544	LG4: 5029485-5035661, complement
Vg-like	Vitellogenin	LOC726783	GB52465	LG2: 10295664-10299129, complement
Vg-like	Uncharacterized LOC726793	LOC726793	GB18024, GB52464	LG2: 10300589-10302675, complement
Vg-like	Uncharacterized LOC411955	LOC411955	GB18247, GB50749	LG2: 6268363-6275046, complement
Hex	Hexamerin 70a	Hex70a	hex71, GB51698	LG8: 11848693-11852800, complement
Hex	Hexamerin 70b; larval storage protein	HEX70b	GB10869, GB51697	LG8: 11855314-11859108, complement
Hex	Hexamerin 70c	Hex70c	GB13613, GB51696; hexamerin	LG8: 11864211-11868236, complement
Hex	Hexamerin 110; high Glx storage protein	Hex110	GB14361, GB44996	LG11: 13517503-13521591, complement

S.I. Table 7. Genes related to epigenetics.

Group	Description	Gene name	Aliases	Location
Dnmt1	DNA (cytosine-5)-methyltransferase PliMCI; DNA methyltransferase 1.1; DNA methyltransferase 1b	LOC726540	Dnmt1b, Dnmt1.1; GB48403	LG10: 6480954-6486997, complement
Dnmt1	DNA methyltransferase 1-associated protein 1	DMAP1	GB45528, LOC408575	LG1: 20275914-20277955
Dnmt1	DNA methyltransferase 1a	Dnmt1a	GB19865, GB47348	LG10:12033925-12039796
Dnmt2	DNA methyltransferase 2; tRNA (cytosine(38)-C(5))-methyltransferase	LOC410512	Dnmt2, GB54141	LG14: 7721772-7723374
Dnmt3	DNA methyltransferase 3; XP_026302146.1; XP_026302147.1; XP_026302148.1; XP_026302149.1; XP_026302150.1	Dnmt3	GB55485	LG2: 15816211-15827587
HAT	Histone acetyltransferase p300-like; CREB-binding protein-like; histone acetyltransferase p300-like	LOC726280	GB12228, GB55583	LG2: 14819581-14835623, complement
HAT	Enoki mushroom; histone acetyltransferase KAT6B	enok	GB41680, LOC724159	LG14: 10578629-10597443, complement
HAT	Males absent on the first; histone acetyltransferase KAT8	mof	GB41293, LOC552696	LG5: 12708838-12711366
HAT	Lysine acetyltransferase chameau ; histone acetyltransferase KAT7; chameau	chm	LOC408793, GB44187	LG4: 11145328-11168624
HAT	Histone acetyltransferase Tip60	Tip60	LOC413101, GB44383	LG10: 3827349-3831915
HAT	Histone acetyltransferase 1; histone acetyltransferase type B catalytic subunit	Hat1	GB46742, LOC552748	LG5: 6593927-6596553, complement
HAT	K(lysine) acetyltransferase 2A; NP_001229397.1; XP_006557745.2; XP_016772396.1; XP_026301489.1	KAT2A	GB19492, GB54338	LG16: 1285857-1289377, complement
HDAC	Histone deacetylase 6	HDAC6	GB42847, LOC725938	LG9: 7890969-7898580
HDAC	Histone deacetylase 4; histone deacetylase 5	HDAC4	GB43234, LOC408331	LG11: 16219663-16243418
HDAC	SIN3-associated polypeptide 30; histone deacetylase complex subunit SAP30 homolog	Sap30	GB47834, LOC551831	LG5: 12046544-12048418
HDAC	Histone deacetylase 3	HDAC3	GB43894, HD3, LOC412350	LG8: 6575527-6577323, complement
HDAC	Histone deacetylase complex subunit SAP130-A	LOC413821	GB42681	LG9: 7510346-7514584, complement
HDAC	Histone deacetylase 1; histone deacetylase Rpd3	HDAC1	GB53438, Rpd3, LOC411503	LG9: 10901790-10906629, complement
HDAC	Sin3 histone deacetylase corepressor complex component SDS3	LOC409995	GB43873	LG8: 6986710-6988684, complement
HDAC	NAD-dependent protein deacetylase Sirt2; NAD-dependent protein deacetylase sirtuin-2; Sirt2 histone deacetylase	LOC409532	GB54259, Sirt2	LG10: 8107574-8110655
HDAC	histone deacetylase complex subunit SAP18	Bin1	GB52889, LOC100576967	NW_020555859.1: 300604-301615
HDAC	NAD-dependent protein deacylase Sirt4; Sirt4 histone deacetylase	LOC551260	GB46254, Sirt4	LG7: 1481264-1482615
HDAC	NAD-dependent protein deacetylase sirtuin-7; Sirt7 histone deacetylase	LOC413728	GB51465, Sirt7	LG11: 5029920-5032169
HDAC	NAD-dependent protein deacetylase sirtuin-1; Sir2 histone deacetylase	LOC411917	GB53035, Sir2	LG4: 12701383-12709086, complement
HDAC	NAD-dependent protein deacylase sirtuin-5, mitochondrial; Sirt5 histone deacetylase	LOC552701	GB52695, Sirt5	LG14: 8228328-8230305
HDAC	NAD-dependent protein deacetylase Sirt6; Sirt6 histone deacetylase	LOC412846	GB51490, Sirt6	LG3: 4701410-4705235
HDAC	Histone deacetylase 6	LOC727620	GB48093	Not in annotation release Amel_HAv3.1
